# Biomaterials-Mediated Regulation of Macrophage Cell Fate

**DOI:** 10.3389/fbioe.2020.609297

**Published:** 2020-12-11

**Authors:** Yining Liu, Tatiana Segura

**Affiliations:** ^1^Department of Biomedical Engineering, Duke University, Durham, NC, United States; ^2^Department of Neurology, Duke University, Durham, NC, United States; ^3^Department of Dermatology, Duke University, Durham, NC, United States

**Keywords:** macrophage, biomaterial, immunomodulation, regeneration, wound healing, phenotype, macrophage polarization, mechanotransduction

## Abstract

Endogenous regeneration aims to rebuild and reinstate tissue function through enlisting natural self-repairing processes. Promoting endogenous regeneration by reducing tissue-damaging inflammatory responses while reinforcing self-resolving inflammatory processes is gaining popularity. In this approach, the immune system is recruited as the principal player to deposit a pro-reparative matrix and secrete pro-regenerative cytokines and growth factors. The natural wound healing cascade involves many immune system players (neutrophils, macrophages, T cells, B cells, etc.) that are likely to play important and indispensable roles in endogenous regeneration. These cells support both the innate and adaptive arms of the immune system and collectively orchestrate host responses to tissue damage. As the early responders during the innate immune response, macrophages have been studied for decades in the context of inflammatory and foreign body responses and were often considered a cell type to be avoided. The view on macrophages has evolved and it is now understood that macrophages should be directly engaged, and their phenotype modulated, to guide the timely transition of the immune response and reparative environment. One way to achieve this is to design immunomodulating biomaterials that can be placed where endogenous regeneration is desired and actively direct macrophage polarization. Upon encountering these biomaterials, macrophages are trained to perform more pro-regenerative roles and generate the appropriate environment for later stages of regeneration since they bridge the innate immune response and the adaptive immune response. This new design paradigm necessitates the understanding of how material design elicits differential macrophage phenotype activation. This review is focused on the macrophage-material interaction and how to engineer biomaterials to steer macrophage phenotypes for better tissue regeneration.

## Introduction

**Figure 1:**
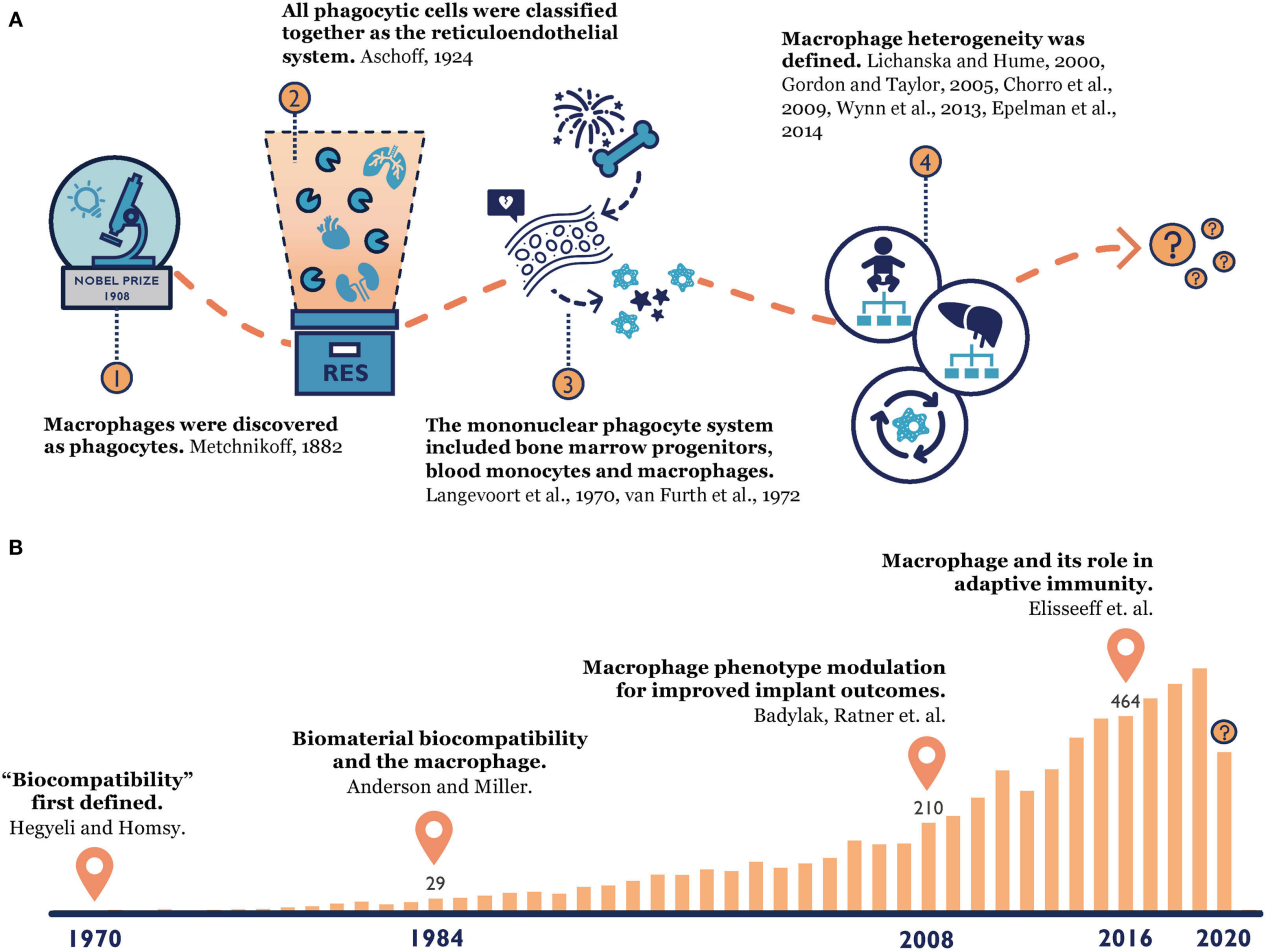
**(A)** Macrophage origin: from the time Metchnikoff discovered macrophages (Metchnikoff, 1883), to the establishment of the reticuloendothelial system (Aschoff, 1924) and mononuclear phagocyte system (Langevoort, 1970; van Furth et al., 1972a), to the current consensus of macrophage heterogeneity (Lichanska and Hume, 2000; Gordon and Taylor, 2005; Chorro et al., 2009; Wynn et al., 2013; Epelman et al., 2014). **(B)** Macrophage-biomaterial response: a PubMed search with key words “macrophage” and “biomaterial” reveals the increasing popularity of this topic over time and our constantly evolving knowledge (Homsy, 1970; Anderson and Miller, 1984; Badylak et al., 2008; Madden et al., 2010; Sadtler et al., 2016).

Our knowledge of macrophages has a long-standing history (Figure 1). When macrophages were first discovered by Élie Metchnikoff, a Russian zoologist, in 1882, they were described as phagocytes that could accumulate at the point of inflammation and clear out invading pathogens (Metchnikoff, 1883). Metchnikoff identified a close connection between the mononuclear phagocytic cells in the spleen, lymph nodes, bone marrow and connective tissue, and he grouped them under the term “macrophage system” (van Furth et al., 1972b). This observation and the later founded phagocytosis theory laid a foundation for innate immunity (Metchnikoff, 1907; Tauber, 2003; Gordon, 2008; Underhill et al., 2016), which earned Metchnikoff a Nobel Prize in 1908. Since then, our understanding of macrophage origin, classification and function continues to evolve. In 1924, Acshoff developed the concept of the reticuloendothelial system (RES) to describe macrophages and other phagocytic cells (Aschoff, 1924). This concept was criticized for only considering cell function rather than their origins, and it was later replaced by the ontogeny-based term “mononuclear phagocyte system (MPS)” in 1969 (Langevoort, 1970; van Furth et al., 1972b; Van Furth, 1980). This widely accepted MPS model proposed that tissue macrophages were terminally differentiated from bone marrow progenitors and blood monocytes (Langevoort, 1970; van Furth et al., 1972b; Van Furth, 1980). However, toward the end of the 20th century, mounting evidence suggested that certain tissue macrophages could proliferate locally (Sawyer et al., 1982; Czernielewski and Demarchez, 1987; Ajami et al., 2007; Chorro et al., 2009). Instead of originating only from bone marrow, researchers found that most tissue macrophages' existence can be traced back to the embryonic stage (Chorro et al., 2009; Epelman et al., 2014). Therefore, macrophage heterogeneity was established and explored further over the last two decades, and hypotheses of macrophages' divergent origins started to prevail (Lichanska and Hume, 2000; Gordon and Taylor, 2005; Wynn et al., 2013). Still, many questions about macrophages have yet to be answered, such as the contribution of recruited monocyte-derived macrophages in the replenishment of tissue-resident macrophages and the functional differences between these two populations during an inflammatory response (Gordon and Taylor, 2005).

Macrophages play a pivotal role in tissue regeneration during injuries and diseases (Wynn et al., 2013). They coordinate with the rest of the immune system to create a pro-regenerative niche at the diseased site, and they recruit progenitor cells to support and promote healing (Martin and Leibovich, 2005; Eming et al., 2014). For instance, during the early stage of acute wound healing, macrophages work side by side with the other innate immune cells (e.g., neutrophils) to debride the wound and construct a provisional matrix (Eming et al., 2014). Together, these effector cells produce chemokines and growth factors to mobilize mesenchymal stem cells, fibroblasts as well as keratinocytes to restore the tissue (Mantovani et al., 2004). However, this endogenous regenerative ability diminishes with age and can also be disrupted in pathological conditions (Wells and Watt, 2018). In the case of diabetes, patients develop non-healing wounds because of the dysregulation in macrophage function, which leads to a perpetuating inflammatory environment and prevents reparative cell infiltration (Eming et al., 2014). These wounds have malfunctioning local milieus that deviate from those of healthy individuals' in both biochemical components (e.g., accumulation of inflammatory cytokines) and mechanical properties (e.g., degraded extracellular matrix) (Schultz and Wysocki, 2009; Christman, 2019). To repair damaged tissue by salvaging the body's natural healing ability, one emerging approach uses immunomodulatory biomaterials to promote immune-mediated tissue regeneration (Rice et al., 2013; Yu et al., 2016b). These materials are designed to actively engage the immune system and manipulate the infiltrating cells, especially macrophages, to perform regenerative functions. This immunomodulatory method thereby reconstructs a local pro-reparative niche and lays down a foundation for endogenous regeneration.

In recent years, more and more research points to macrophages' parts in bridging innate immunity with adaptive immunity and how this bridging role can be leveraged by immunomodulating biomaterials for promoting endogenous repair (Sadtler et al., 2016; Wolf et al., 2019). To this end, a comprehensive understanding of macrophage-biomaterial response is essential. Many excellent reviews cover macrophage mechanotransduction (McWhorter et al., 2015; Mennens et al., 2017; Adams et al., 2019; Jain et al., 2019; Meli et al., 2019; Gruber and Leifer, 2020) and its biomedical applications (Brown et al., 2014; Springer and Fischbach, 2016; Andorko and Jewell, 2017; Spiller and Koh, 2017; Li J. et al., 2018) from various angles. However, no existing review articles provide a current view of macrophage-material interaction from a practical bioengineering perspective. In this review, we synthesize the up-to-date understanding of macrophage biology and macrophage-material response. We also present a pragmatic guidebook for researchers to design biomaterials that can guide context-dependent macrophage polarization for optimal endogenous tissue regeneration.

## Macrophage Heterogeneity and Plasticity

Macrophages are highly heterogeneous immune cells with great diversity in lineages, anatomical distribution, and functional subsets (Wynn et al., 2013). The idea that maintenance of tissue-resident macrophages in mice relies on the recruitment and differentiation of blood monocytes was once mainstream (van Furth et al., 1972b; Wynn et al., 2013; Ginhoux and Jung, 2014). However, recent fate-mapping studies challenged this idea by comprehensively demonstrating that most tissue-resident macrophages are derived from yolk sacs and fetal livers in the embryonic stage, and they can self-renew to persist into adulthood independent of monocytes (Ginhoux et al., 2010; Schulz et al., 2012; Hashimoto et al., 2013; Wynn et al., 2013; Yona et al., 2013). Only some highly specialized macrophage populations, such as those in the skin, intestine, and splenic marginal zone, require continuous repopulation by bone-marrow-derived precursors (Schulz et al., 2012; Tamoutounour et al., 2013; Yona et al., 2013; Bain et al., 2014). This dual origin theory of tissue macrophages also holds true in humans (Bajpai et al., 2018).

Macrophage plasticity is another hallmark that supports multifaceted roles for macrophages in different tissues, organs and disease states (Mosser and Edwards, 2008). Besides the control of intrinsic differentiation pathways, macrophages are also subject to the influence of the local microenvironment and perform context-based functions (Gosselin et al., 2014; Lavin et al., 2014; Wills et al., 2017). For example, when peritoneal macrophages were engrafted into donor lungs, these macrophages upregulated lung macrophage-specific genes, demonstrating that differentiated tissue-resident macrophages retain their plasticity and that local tissue cues can alter macrophage function (Lavin et al., 2014). However, while macrophage plasticity can dictate its function, it is also possible to have functionally and developmentally distinct macrophage populations coexisting in the same tissue, such as large and small peritoneal macrophages (Ghosn et al., 2010). Therefore, when designing biomaterials to engage macrophages, it is essential to characterize macrophages with a comprehensive paradigm that takes into account their origin, plasticity, and overall heterogeneity within tissues.

**Figure 2:**
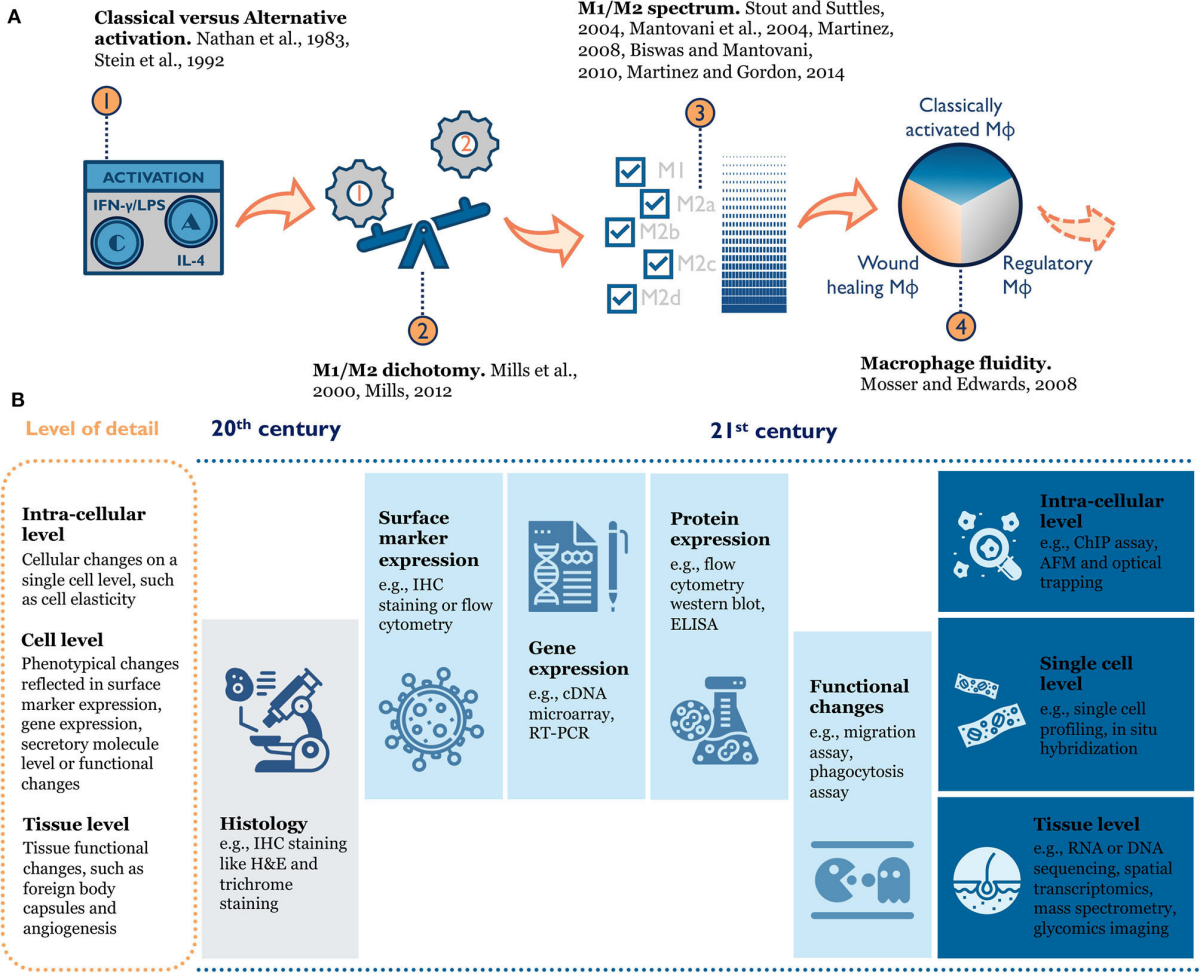
**(A)** Evolution of macrophage phenotype models: from the historical M1/M2 (Classical vs. Alternative) dichotomy (Nathan et al., 1983; Stein et al., 1992; Mills et al., 2000; Mills, 2012), to an M1/M2 spectrum with multiple M2 sub-categories (Mantovani et al., 2004; Stout and Suttles, 2004; Martinez et al., 2008; Biswas and Mantovani, 2010; Martinez and Gordon, 2014), to the macrophage color wheel model (Mosser and Edwards, 2008), there is an increasing appreciation for the complexity of macrophage phenotype. **(B)** Rapid improvement in tools to characterize macrophage phenotype enables researchers to better understand macrophage biology in the context of tissue or materials. IHC, immunohistochemistry; RT-PCR, Real time polymerase chain reaction; ELISA, enzyme-linked immunosorbent assay; ChIP, chromatin immunoprecipitation; AFM, atomic force microscopy.

Macrophage phenotype is difficult to define because of inherent heterogeneity in this cell population and their plasticity in response to environmental changes (Figure 2). When researchers first tried to understand macrophage phenotype, a modular approach mirroring T helper type 1 and T helper type 2 polarization was used to divide macrophage phenotypes into M1 and M2 (classical activation and alternative activation) (Nathan et al., 1983; Stein et al., 1992; Mills et al., 2000; Mills, 2012, 2015). This paradigm was useful for early *in vitro* study as it clearly defined the activation cytokines and expected functional changes (e.g., surface receptors, ligands, produced cytokines) for each phenotype. As more evidence accumulated, a few disparate macrophage phenotypes emerged that could not simply be grouped into “M2” (Edwards et al., 2006). Therefore, the M1/M2 dichotomy was further expanded to include subcategories like M2a, M2b, and M2c (Mantovani et al., 2004; Martinez et al., 2008; Biswas and Mantovani, 2010). This categorical view still ran the risk of oversimplifying the intricate population of macrophages by force-fitting them into defined categories. Over the past decade, scientists conducting epigenetics, gene expression, and functional studies discovered new macrophage phenotypes that the traditional M1/M2 model failed to characterize (e.g., tumor-associated macrophages) (Mosser and Edwards, 2008; Xue et al., 2014; Malyshev and Malyshev, 2015). Thus, a color wheel model with different “shades” of activation was proposed to account for both fundamental functions and the context-specific roles macrophages perform (Mosser and Edwards, 2008; Ginhoux et al., 2016). In other words, macrophage phenotype should be seen as a dynamic and not a static process (Mosser and Edwards, 2008; Wills et al., 2017).

Given the fluidity of macrophage phenotype and the plasticity of the cells, it's very hard to both accurately define them and comprehensively characterize them. As with many biological assays, common tools used to study macrophage phenotype only capture a snapshot at the moment of sampling. In reality, macrophages are constantly adapting to the changing environment and their phenotypes should not be viewed as an end-point definition (Mosser and Edwards, 2008). A universal language on how to define macrophages has yet to be widely accepted and adopted (Murray et al., 2014), especially in *in vivo* settings because of the many factors at play. For example, macrophages in wounds exhibit a complicated phenotype with features found in both M1 and M2 macrophages (Daley et al., 2010; Novak and Koh, 2013). Thus, the term “macrophage phenotype” and the phenotype definitions should be considered with careful deliberation. When researchers refer to phenotype results from different studies or report their findings, a multi-aspect description needs to be provided to capture the full picture of activated macrophages, such as the origin of the cells, the systemic and local milieu, the combination of markers and functions they share, and the timing of activation.

## A Double Edge Sword *in vivo*

Macrophages are central to many disease stages and serve multifaceted roles during physiological and pathological processes (Gordon, 1995). Because of their plasticity, macrophages are highly susceptive to environmental stimuli and they act as a double edge sword *in vivo* (Wynn et al., 2013). In a normal, healthy adult, macrophages play integral roles in maintaining tissue homeostasis, inflammation and repair. On one hand, they are the diligent “janitors,” clearing out dead cells and extraneous cellular debris as part of the regular metabolic process (Mosser and Edwards, 2008). Distinct tissue-resident macrophages perform tissue-specific homeostatic functions, such as the clearance of apoptotic neutrophils and erythrocytes in the spleen and liver. If a timely removal of these cells by macrophages fails, severe results ensue, such as neutropenia, splenomegaly, and reduced body weight (Gordy et al., 2011). On the other hand, macrophages act as the primary sensor of danger signals and the first responders in the innate immune system for host defense (Gordon, 1995). In the case of acute wound healing, macrophages govern the inflammation stage and orchestrate regeneration (Leibovich and Ross, 1975). Within minutes of injury, tissue-resident macrophages recognize danger signals, like damage-associated molecular patterns, and help initiate the local inflammatory response (Minutti et al., 2016). Monocyte-derived macrophages are then recruited to the wound by the local presence of inflammatory chemokines and cytokines, such as monocyte chemoattractant protein-1 (MCP-1), tumor necrosis factor-alpha (TNF-α), and interferon-gamma (IFN-γ), and they further amplify the inflammatory response (Krzyszczyk et al., 2018). These new-comers actively attempt to phagocytose foreign materials and produce proteases (e.g., matrix metalloproteinases/MMPs) to break down the damaged matrix (Ginhoux and Jung, 2014). They also secrete various factors (e.g., chemokines, cytokines) to coordinate support cells and assist tissue reconstruction. Selective depletion of macrophages in mice during the inflammatory phase impairs wound vascularization and contraction (Mirza et al., 2009; Lucas et al., 2010). Conversely, removing macrophages during the tissue formation phase leads to severe hemorrhage in wound tissues (Mirza et al., 2009; Lucas et al., 2010). Both cases further demonstrate that macrophages are central players in wound healing. When pathological conditions occur, macrophages' homeostatic and reparative functions can be overridden, which has led to a causal association of macrophages with many diseases.

Disturbances in macrophage function contribute to a broad spectrum of pathologies, such as cancer and inflammatory disorders (Wynn et al., 2013). For diabetes patients, the underlying pathologies, like elevated glucose, are believed to promote a pro-inflammatory macrophage phenotype (Wen et al., 2006; Mirza and Koh, 2011; Bannon et al., 2013). These cells accumulate in the wound bed and contribute to the uncontrolled production of pro-inflammatory cytokines, chemokines and proteases, as well as growth factors (Eming et al., 2010). An overabundance of MMPs, as an example, can break down critical extracellular matrix (ECM) proteins and prevent new tissue formation (Wysocki et al., 1993). Overall, the imbalance of these key molecules, like pro-inflammatory cytokines, forms a vicious cycle by maintaining a macrophage pro-inflammatory phenotype and preventing regenerative cell infiltration. A predominance of pro-inflammatory macrophages is considered a major hallmark for non-healing wounds, and restoring the properly-regulated transition in macrophage phenotypes remains key to the development of a potential solution (Willenborg and Eming, 2014).

Macrophage-directed therapies are promising for the treatment of a range of diseases (Springer and Fischbach, 2016; Spiller and Koh, 2017; Li R. et al., 2018). Generally, the treatments can be categorized into 4 types: (1) exogenous macrophage supplementation; (2) delivery of molecules to modulate endogenous macrophage phenotypes or alter their numbers; (3) delivery of biomaterials to modulate endogenous macrophage phenotypes; (4) a selective combination of 1-3. The second approach has been most studied. Researchers using this approach delivered key molecules (e.g., recombinant interleukin-4/IL-4, TNF-α neutralizing antibody) to recruit endogenous macrophages and induce their pro-regenerative function or block pro-inflammatory signaling pathways or products (Salmon-Ehr et al., 2000; Goren et al., 2007). However, several disadvantages have arisen with these methods. Given that the details regarding timing and specific roles of each macrophage phenotype are still yet to be fully elucidated, an optimal dosage to elicit desired phenotypes in a timely manner is hard to achieve. Additionally, the delivery of molecules or cells directly to the wound bed also ignores the importance of the microenvironmental context in shaping macrophage responses (Gosselin et al., 2014; Lavin et al., 2014). In a pro-inflammatory milieu, excessive amounts of proteases might expedite the degradation of active molecules, and exogenous activated M2 macrophages could be skewed to perform M1 function. To achieve long-lasting healing outcomes, it is crucial to first address the aberrant microenvironment. A combined method, with immunomodulatory biomaterials to reconstruct the local environment, will be better suited to fine-tune macrophage response and achieve better regeneration.

## Macrophage-Material Response: What Do We Know and What Should We Do?

**Figure 3:**
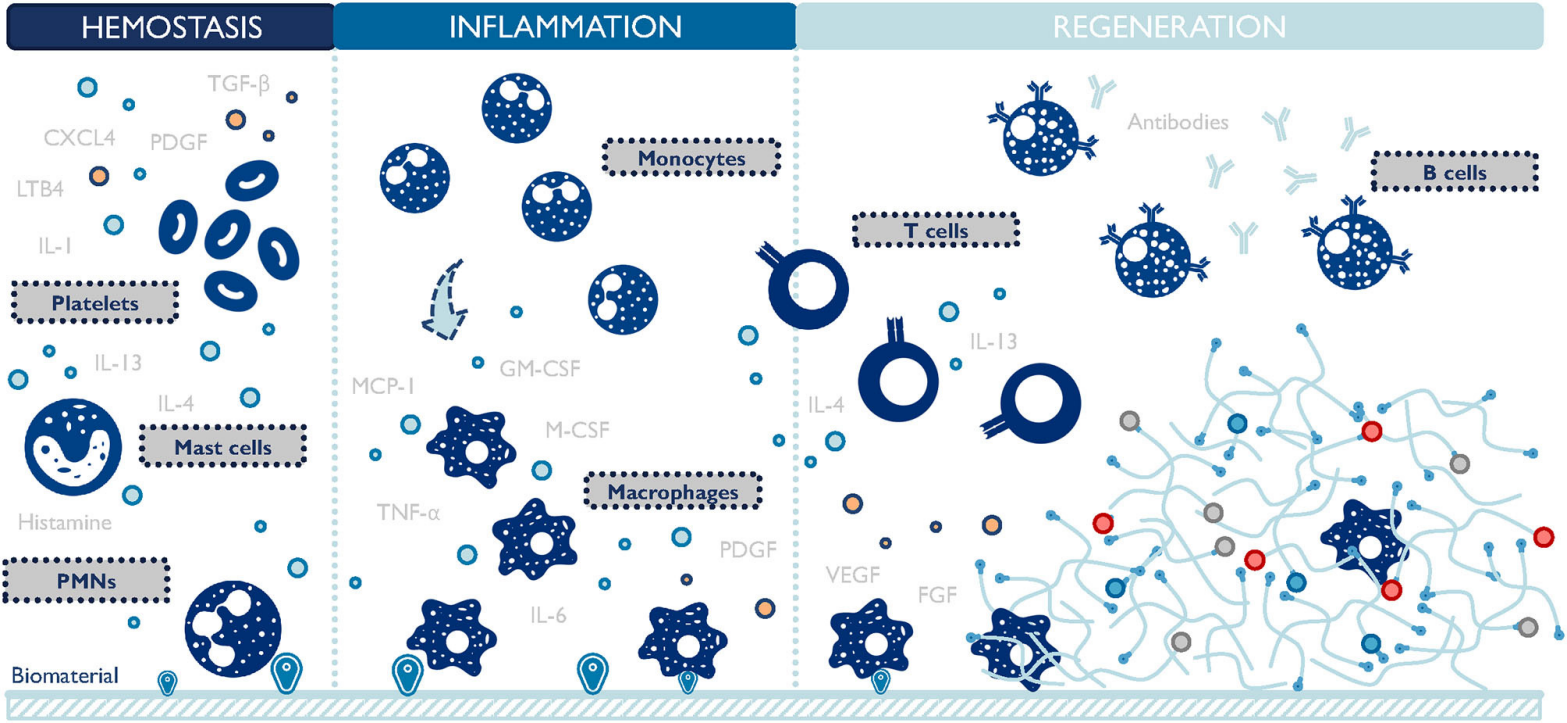
Macrophages are central players during the host response to biomaterials. They closely interact with other cells and persist at the material-body interface throughout the inflammation and regeneration stages. TGF-β, transforming growth factor-beta; PDGF, platelet-derived growth factor; CXCL4, chemokine (C-X-C motif) ligand 4; LTB4, leukotriene B4; IL, interleukins; PMNs, polymorphonuclear leukocytes; TNF-α, tumor necrosis factor-alpha; M-CSF, macrophage-colony stimulating factor; GM-CSF, granulocyte macrophage colony stimulating factor; MCP-1, monocyte chemoattractant protein-1; VEGF, vascular endothelial growth factor; FGF, fibroblast growth factor. The list of molecules is not comprehensive.

The design of biomaterial scaffolds that are capable of modulating the local microenvironment requires a closer look at the macrophage-material interaction and the resulting changes in macrophage phenotype. This interaction has been studied extensively in the context of foreign body response since the early 1970s (Coleman et al., 1974). A foreign body response (FBR) is characterized by the persisting existence of immune cells, particularly macrophages, and the encapsulation of the implanted material by fibrotic tissues (Anderson et al., 2008). Macrophages oversee the inflammation process of the host reaction to implants (Figure 3). They function to clear out debris and foreign materials via phagocytosis, produce enzymes to remodel the provisional matrix, and secrete signaling molecules to recruit support cells, such as fibroblasts. In an attempt to minimize the impact of a foreign implant on the body, macrophages fuse into foreign body giant cells (FBGC), and actively seek to degrade the implant. If this process fails, FBGCs work with fibroblasts to deposit collagen layers and wall off the implant by forming a dense fibrotic capsule around it (Anderson et al., 2008). FBR can render implanted biomaterials or medical devices non-functional by preventing drug release, reducing blood supply, and causing contraction and pain. Surprisingly, even now it is still unclear which macrophage phenotype plays a major role in initiating FBR. M1 macrophages are closely related to inflammation and reduced M1 presence has been observed to attenuate FBR (Goreish et al., 2004). In addition, multiple studies proved that a higher M2 to M1 ratio was associated with more constructive remodeling and implant vascularization (Badylak et al., 2008; Brown et al., 2012; Spiller et al., 2014). Conversely, immunoregulatory cytokines (e.g., platelet-derived growth factor/PDGF, transforming growth factor-beta/TGF-β), inflammatory cytokines (e.g., TNF-α), and chemokines (e.g., MCP-1) are implicated in the formation of FBR, connecting both M1 and M2 macrophages to this process (Kao et al., 1995; Hernandez-Pando et al., 2000; Gretzer et al., 2006; Rodriguez et al., 2009). Taken together, these results suggest that either both M1 and M2 macrophages contribute to FBR collaboratively, or a hybrid M1-M2 phenotype exists throughout FBR. The latter hypothesis is further proof that the M1/M2 dichotomy falls short in accurately categorizing macrophage phenotypes.

Given the impact of FBR on implants, traditional strategies for biomaterial design focus on evading or suppressing inflammation, especially macrophage response, in order to mitigate FBR. For example, surface hydrophilicity can be used to overcome non-specific protein absorption and reduce FBGC formation (Quinn et al., 1997; Jenney and Anderson, 1999; Voskerician et al., 2003; Collier et al., 2004). However, growing evidence has shown that macrophage engagement during implantation can be harnessed to improve implant success rates (Spiller et al., 2014; Yu et al., 2016a) and that a timely transition from M1 to M2 phenotype benefits tissue remodeling (Badylak et al., 2008; Brown et al., 2012; Spiller et al., 2014; Yu et al., 2016a; Witherel et al., 2020). Many approaches to promote this M1-M2 shift, like a sequential delivery of immunomodulatory cytokines IFN-γ and IL-4, have achieved some positive outcomes (Mokarram et al., 2012; Spiller et al., 2015). This evolving knowledge base of macrophage-material interaction has contributed to a new era of immune-modulating materials where material design is centered around desired immune responses.

During macrophage-material interaction *in vivo*, the material itself acts as a temporary niche for macrophages to reside in, and the properties of the material weigh in on macrophage phenotypes. As discussed in the previous section, macrophages are known to adapt to microenvironmental features, such as biochemical and physical signals. The molecular mechanisms behind common soluble factors, like IL-4 and lipopolysaccharides (LPS), have been extensively studied, while new information continues to emerge with the advance of biotechnologies (Martinez and Gordon, 2014; Ramirez et al., 2017). Remarkably, emerging evidence suggests that long-ignored physical cues play an important modulating role in macrophage activation, but the signaling pathways have yet to be fully elucidated (McWhorter et al., 2015; Jain et al., 2019) (Table 1, refer to Supplementary Table 1 for more detail information). A broad range of material properties, such as pore size (Madden et al., 2010; Garg et al., 2013; Sussman et al., 2014; Wang et al., 2014), shape and geometry (Matlaga et al., 1976; Veiseh et al., 2015), stiffness (Blakney et al., 2012; Sadtler et al., 2019), topography (Chen et al., 2010; Wang et al., 2016; Shayan et al., 2018) and surface modification (e.g., hydrophilicity, integrin engagement) (Brodbeck et al., 2002; Antonov et al., 2011; Blakney et al., 2012; Swartzlander et al., 2015; Cha et al., 2017), have been proven to modulate macrophage behavior and tune implantation outcomes. However, a lot of these studies simply observed correlations between material designs and macrophage phenotypes without exploring further the molecular mechanism behind them. For instance, poly (2-hydroxyethyl methacrylate-co-methacrylic acid) hydrogel scaffolds with pore diameters of 30–40 μm showed maximum vascularization and minimal fibrotic response following implantation (Madden et al., 2010). This outcome was coupled with an increased number of macrophages in the implants expressing both nitric oxide synthase 2 (NOS2, M1 marker) and macrophage mannose receptor (MMR, M2 marker) at the same time (Madden et al., 2010). A similar study using the same scaffold system revealed that the pro-angiogenic 34 μm porous implants had more macrophages accumulating in the pores with a primarily M1 marker expression (NOS2 and IL-1R1) (Sussman et al., 2014). Another work using expanded polytetrafluoroethylene scaffolds demonstrated that larger intranodal distance (4.4 μm) induced a significantly thinner capsule *in vivo* but promoted early proinflammatory cytokine production and gene transcription by monocytes/macrophages *in vitro* (Bota et al., 2010). These studies highlight the potential of using scaffolds with tunable pore sizes to guide macrophage phenotypes, but the results did not consistently point to one particular optimal pore size inducing a preferable macrophage phenotype to achieve desired outcomes. This is partly due to the complex *in vivo* environment and varying experimental settings, such as the different materials and animal models used, and the limited biomarkers selected for macrophage characterization. Ultimately, material design needs to target a clear mechanotransduction pathway of macrophages so that it can be translatable between material systems and disease applications.

**Table 1:** Modulating macrophage phenotype by physical cues.

**Physical cues**	**Rationale**
Stiffness	Stiffness-dependent changes generally include enhanced adhesion and spreading, increased actin and cytoskeletal stiffness, increase in proliferation and migration as well as increased phagocytosis. But the connection between stiffness and macrophage phenotype remains complicated
Surface topography	Surface topography, such as roughness and micropatterns, can guide macrophage behavior by modifying their adhesion, spreading, elongation, and motility on the surface. Specifically, using topography design to force macrophages into elongated cell shape is shown to promote a pro-regenerative M2 phenotype
Surface modification	Surface modification like coating or modifying surface chemistry directly alters how macrophages engage with the material. There is a clear role for integrin-mediated regulation of macrophage migration, phagocytosis, and activation, but the precise mechanisms still remain relatively unknown
Geometry	Scaffold geometry affects macrophage phenotype by spatially confining macrophages and limiting their spreading, thereby leads to an alteration in actin polymerization, chromatin compaction, and epigenetic alterations
Hemodynamic loads	Macrophages reside within mechanically active tissues and are constantly exposed to dynamic external forces, such as stretch and cyclic strain. These forces can cause macrophages to elongate along the direction of force, therefore affecting their phenotypes. However, there is still no consensus on the mechanism of mechanical forces in influencing macrophage function

**Figure 4:**
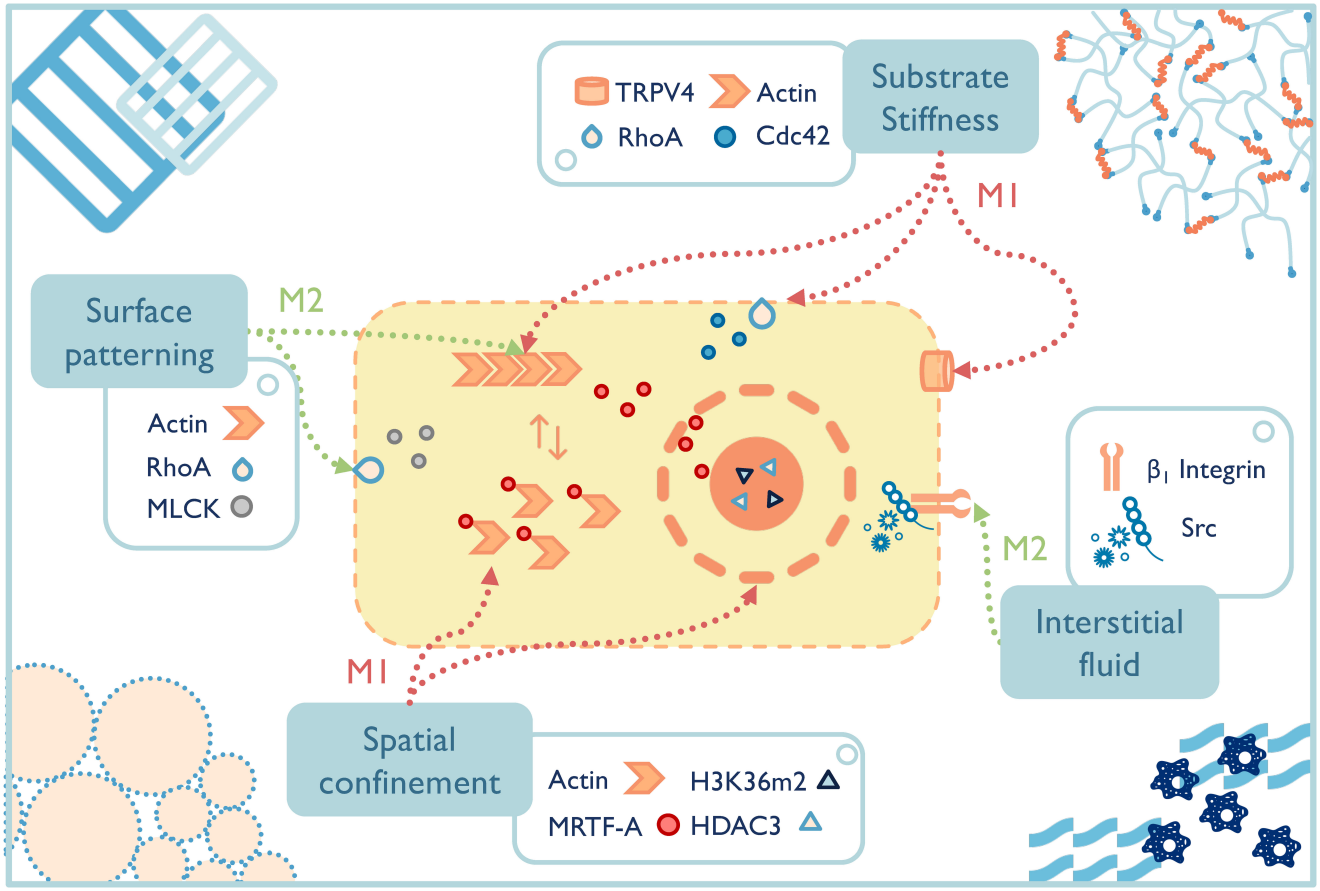
The fate of macrophages can be greatly affected by material properties, both those inherent to the selected materials and the additional engineered functionality. Potential macrophage mechanotransduction pathways have been probed for a few properties, such as surface patterning (McWhorter et al., 2013), substrate stiffness (Patel et al., 2012; Scheraga et al., 2016; Gruber et al., 2018), spatial confinement (Jain and Vogel, 2018), and interstitial fluid (Li R. et al., 2018), while others remain to be elucidated. TRPV4, transient receptor potential cation channel subfamily V member 4; RhoA, ras homolog family member A; Cdc 42, cell division cycle 42; MLCK, myosin light-chain kinase; HDAC, histone deacetylase 3; MRTF-A, myocardin-related transcription factor-A.

In the past two decades, well-defined *in vitro* systems have been employed to more specifically understand the mechanotransduction mechanisms that lead to phenotypic macrophage changes to common mechanical signals (Figure 4). For example, preventing bone marrow-derived macrophages or RAW264.7 cells from spreading by spatial confinement, such as using micropatterned surface, microporous substrates or cell crowding, reduced LPS-stimulated transcriptional programs and cytokine expression (Jain and Vogel, 2018). The study elegantly illustrated that confining macrophages in a small pore limited actin polymerization and thus lowered the nuclear translocation of the actin-dependent transcription co-factor, myocardin-related transcription factor-A, which downregulated the inflammatory response (e.g., less pro-inflammatory cytokine secretion, lower phagocytic potential of macrophages). Spatial constraints also led to the chromatin compaction and epigenetic alterations (e.g., lower histone deacetylase 3 levels, increased H3K36-dimethylation). Although the results of these studies shed light on some potential pathways that guide macrophage response to mechanical stimuli, macrophages cultured on a two-dimensional surface still cannot fully recapitulate macrophages' structures and function in 3D culture or *in vivo* (Van Goethem et al., 2011). Therefore, future studies on mechanotransduction mechanisms of macrophages should turn to 3D systems with a range of independently controlled properties in order to achieve a far-reaching physiological significance.

## Macrophage-Centered Immunomodulatory Biomaterials for Functional Repair

The ultimate purpose of regenerative materials is to restore normal tissue function. This goal cannot be achieved solely by engaging one or two key cell types. To obtain functional recovery, the material must synergize with the systemic and local immune responses, as well as coordinate with the microenvironment and supporting cells. Because macrophages bridge innate immunity and adaptive immunity, a new paradigm was established to harness macrophage responses by immunomodulating biomaterials for endogenous repair (Sadtler et al., 2016; Wolf et al., 2019). A recent study demonstrated that synthetic porous scaffolds eliciting a Th2 adaptive immune response can achieve regenerative healing through a macrophage/IL-33 mechanism (Griffin et al., 2020). It's worth noting that although the specific peptide that this study used was a poor activator of macrophage innate immune signaling *in vitro*, when the peptide was presented in porous scaffolds *in vivo*, an IL-33-related type 2 myeloid cell recruitment and an antigen-specific immunity were induced to support tissue remodeling and skin regeneration. This further illustrated that material design should target the immune system as a whole, rather than one cell type, to deliver an optimal outcome.

**Figure 5:**
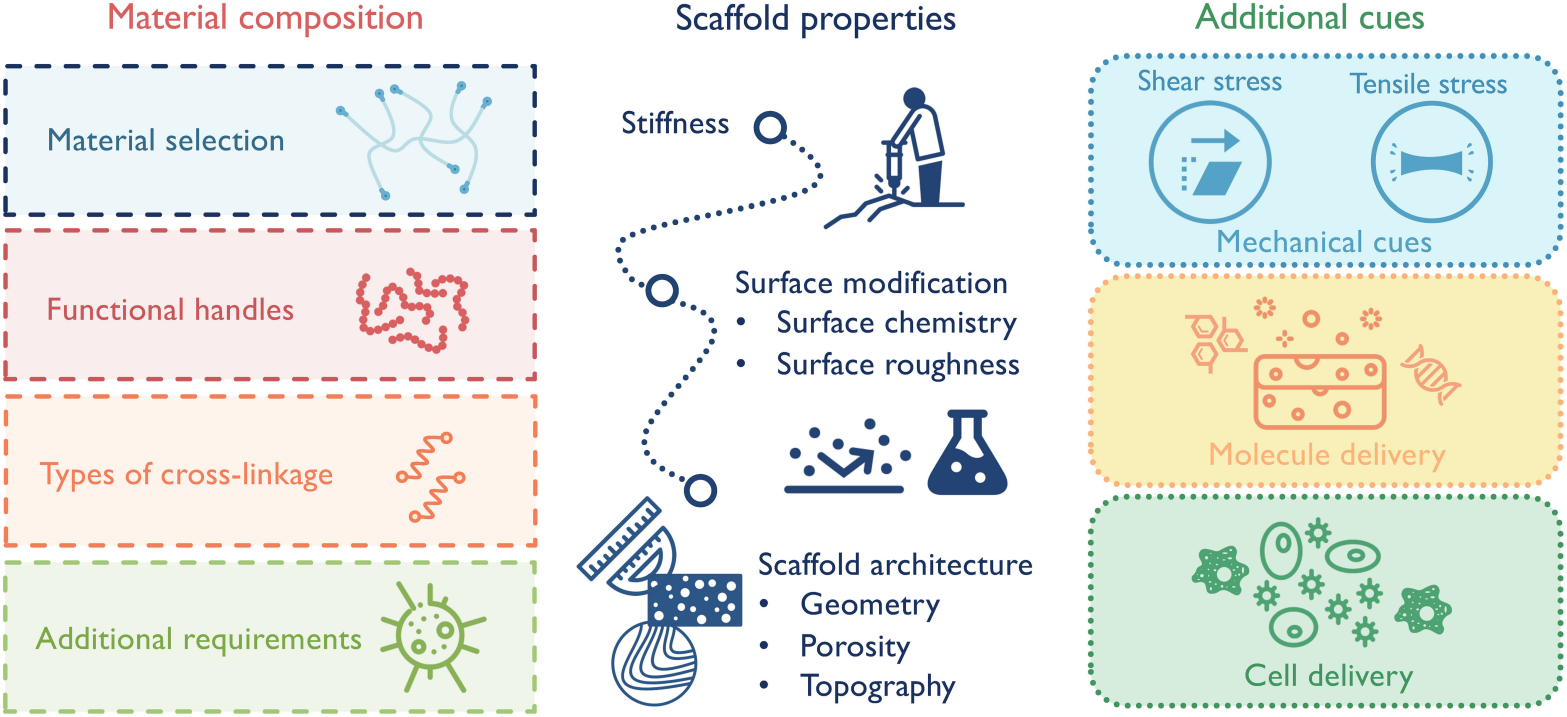
Design principles for macrophage-centered immunomodulatory biomaterials:(1) select material composition that best suits the application; (2) design scaffold properties to elicit desired macrophage-governed inflammatory response; (3) combine with additional cues to maximize therapeutic effect, such as stem cells or nanoparticles.

Macrophages, as key facilitators of functional tissue repair, remain in the center of design for immunomodulatory biomaterials. In order to ensure that material designs achieve the designated goals of promoting the desired macrophage phenotype, material composition (e.g., natural vs. synthetic), scaffold physical properties (e.g., microstructure and viscous vs. elastic mechanical properties), and additional cues (e.g., chemokines, nanoparticles) must be carefully considered (Figure 5). The backbone material sets the cornerstone for the general immune response and the following design options. Naturally derived materials, like ECM components or decellularized tissues, have inherent cell-binding motifs (e.g., integrins, CD44) and can selectively promote a range of immune responses based on their composition (Badylak et al., 2008; Boddupalli et al., 2016; Sadtler et al., 2019). As an example, heparin and other sulfated polysaccharides have the ability to bind growth factors and some cytokines, many of which directly activate macrophages and, thus, dictate their phenotype (Capila and Linhardt, 2002). This ability has been exploited in one study to sequester heparin-binding factors in diabetic wounds to reduce inflammation and promote wound healing (Lohmann et al., 2017). Synthetic materials, such as polyethylene glycol (PEG), have high plasticity for chemical modification and are less immunogenetic. These substrates can serve as “clean slates” to release molecules or present factors spatially and/or temporally (Cha et al., 2017). Functional handles can also be incorporated into the scaffolds to further direct macrophage responses. For instance, different adhesion receptor engagement, like αVβ3 integrin, can be leveraged to alter macrophage phenotype (Kao et al., 2001; Antonov et al., 2011). In terms of scaffold properties, a few parameters impacting macrophage phenotype should be considered, including substrate stiffness, surface hydrophobicity, surface modification, and scaffold microstructure (McWhorter et al., 2015; Jain et al., 2019). While some features can be combined in one scaffold, it is often difficult to separately control mechanical and biochemical properties. For example, in chemically crosslinked hydrogels, the substrate stiffness is tied up with material degradation, ligand density and mesh size. Increasing stiffness by additional cross-linkages also leads to a slower degradation rate, a smaller mesh size/diffusion rate, and an increased local ligand density. Therefore, modulating macrophage response with scaffold stiffness in these systems cannot be easily decoupled with the other confounding factors. Also, when manipulating matrix properties, care should be taken to consider not only macrophage response, but also macrophages' coordination with other cell types to achieve synergistic responses. In the case of scaffold porosity, a smaller pore size may be in favor of M2 macrophage response, but larger pore size could be beneficial for the growth of blood vessels, which in the long run supports the implant success rate (Madden et al., 2010; Feng et al., 2011). Additionally, to avoid any unwanted pro-inflammatory macrophage activation, biomaterials should also be verified that they are substantially free of known toxic or harmful materials, such as endotoxins or residual cellular debris (Lieder et al., 2013).

In our lab, a new class of injectable biomaterials using hydrogel building blocks was designed to improve cellular infiltration and modulate host response (Griffin et al., 2015). A microporous annealed particle (MAP) scaffold is formed by interlinking the particles together, which contains an interconnected network of void space with channels that are on the length-scale of cells. Such a scaffold offers enormous tunability by virtue of its granular nature, where a bottom-up approach to design starts at the individual particle. These building blocks have been fabricated using a variety of synthetic and natural backbone materials (e.g., PEG, hyaluronic acid) with a range of chemical modifications to allow for particle crosslinking, cargo delivery (e.g., growth factor, stem cells, DNA), and to influence cellular behavior (e.g., RGD). For example, particles are frequently engineered with RGD peptides to promote cellular infiltration and migration throughout the scaffold, while the inclusion of growth factors offers additional cues to traversing cells (Truong et al., 2019). The shape, size, and stiffness of the particles composing a MAP scaffold not only dictate bulk mechanical properties of the scaffold, but also set the internal landscape of the void space that is sensed by the cells. By ranging size, stiffness, and RGD concentration, one study was able to develop particles with an optimal combination that, when used as a MAP scaffold, demonstrated superior gene transfection capabilities (Truong et al., 2019). Yet the power of granular biomaterials extends beyond the design of individual particles. Including multiple particle species into a single scaffold can offer a higher level of material tunability. By incorporating particle heterogeneity, a scaffold may serve more than one primary function, such as promoting stem cell growth while simultaneously inhibiting bacterial growth (Cai et al., 2018). MAP scaffolds can also be designed with spatial heterogeneity, where physical or chemical gradients can be maintained during injection due to the jamming properties of granular materials (Darling et al., 2018; Riley et al., 2019). Remarkably, MAP gels have already demonstrated great promises in promoting functional tissue repair in wound healing and stroke with a reduced inflammatory response (e.g., reduced CD11b+ immune cell infiltration) (Griffin et al., 2015, 2020; Sideris et al., 2016; Nih et al., 2017; Darling et al., 2020). Specifically, in stroke infarct areas filled with MAP gels, there is a much higher ratio of infiltrating pro-reparative arginase 1+ (Arg-1+) macrophage comparing to no treatment control (Sideris et al., 2019). Taken together, these results supported that MAP scaffold is not only appealing as a tool for elucidating the mechanotransduction pathways of macrophages, but also as a potent immunomodulatory platform for macrophage-targeting therapies.

## A Look Into the Future

Modern biomaterials continue to emerge and evolve, unlocking infinite potentials for tailoring macrophage-centered therapies. These materials are promising because they can offer a controlled and tunable microenvironment, incorporating mechanical, and biochemical properties, as well as their temporal and spatial presentation. Although research on new biomaterials and material designs for immunomodulation are burgeoning in recent years, a refocus on directing macrophage behavior to achieve suitable immune responses needs to be emphasized. An optimal biomaterial design should act synergistically with additional mechanical cues, molecules, or cells (that are delivered or found endogenously) to coax macrophages into pro-regenerative phenotypes. These macrophages, together with tissue-resident cells and recruited immune cells, can collectively orchestrate the regenerative process.

There is still much to be learned about macrophages and their roles in endogenous repair, such as the different contribution of tissue-resident macrophages and their bone marrow-derived counterparts during healing and host responses. A common nomenclature of dynamic macrophage phenotype, taking into account both macrophages' heterogeneity and plasticity, should also be unified to benefit both *in vitro* and *in vivo* studies. Currently, the investigation of macrophage mechanotransduction pathways remains an important area of research, as it can direct new material design strategies to harness macrophage activity for endogenous tissue regeneration and disease treatment. Armed with the booming knowledge of macrophage and macrophage-material response, the next generation of macrophage-centered immunomodulatory biomaterials should be able to conquer broader land and achieve more and more successful translation into clinical settings.

## Author Contributions

YL is credited with the conception and writing of the manuscript as well as the creation of figures. TS provided valuable edits, suggestions, and feedback. All authors contributed to the article and approved the submitted version.

## Conflict of Interest

The authors declare that the research was conducted in the absence of any commercial or financial relationships that could be construed as a potential conflict of interest.
