# Supplementary material for: Biomaterials-Mediated Regulation of Macrophage Cell Fate
Source: Front Bioeng Biotechnol. 2020 Dec 11;8:609297. doi: 10.3389/fbioe.2020.609297 (PMC7759630; doi:10.3389/fbioe.2020.609297)
Supplement: Supplementary file 1 [file Table_1.docx]

Biomaterials-mediated regulation of macrophage cell fate

Yining ****Liu^1^, Tatiana Segura^1-3*^, PhD****

**^1^Department of Biomedical Engineering, Duke University, Durham, NC 27708, USA**

**^2^Department of Neurology, Duke University, Durham, NC 27708, USA**

**^3^Department of Dermatology, Duke University, Durham, NC 27708, USA**

*** Corresponding author:**Tatiana Segura, PhD
[Tatiana.segura@duke.edu](mailto:Tatiana.segura@duke.edu)

Keywords: Macrophage, Biomaterial, Immunomodulation, Regeneration, Wound healing, Phenotype, Macrophage polarization, Mechanotransduction

**Supplementary Table 1 Selected studies on physical and mechanical regulation of macrophage phenotype using biomaterials**

| Mechanical cues | Materials | Cell-binding domain | Model | Macrophage characterization | | | | Outcomes | Ref. |
| --- | --- | --- | --- | --- | --- | --- | --- | --- | --- |
|  |  |  |  | Surface marker | Gene expression | Protein expression | Functional change |  |  |
| Stiffness (1.4, 10, 348 kPa) | Polyacrylamide | RGD | THP-1/2D |  |  | 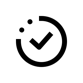 |  | Cells secreted most TNF-α on 1.4 kPa substrate and least on 348 kPa after normalizing the bulk population secretion to the number of adherent cells. The IL-8 secretion profile appeared to be biphasic in that secretion was low on 1.4 kPa and 348 kPa but high on the intermediate 10 kPa substrate. | (Irwin et al., 2008) |
| Stiffness (130, 240, 840 kPa) | Poly(ethylene glycol) diacrylate | RGD | RAW 264.7/2D |  |  | 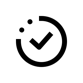 |  | When stimulated with LPS, the increased expression of TNF-α, IL-1β, and IL-6 was significantly reduced with the softest hydrogels. | (Blakney et al., 2012) |
|  |  |  | Subcutaneous implantation in mice |  |  |  | 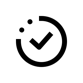 | 28 days post-implantation the layer of macrophages at the implant surface was significantly lower in the softest hydrogels. |  |
| Stiffness (1.2, 140 kPa) | Polyacrylamide | Poly-L-lysine | RAW 264.7, U937 and primary human alveolar macrophages/2D |  | 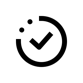 | 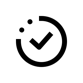 | 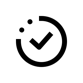 | Macrophages cultured on the more rigid substrate exhibited increased phagocytosis of both unopsonized and IgG opsonized latex beads. Production of reactive oxygen species also increased with increasing substrate rigidity. With LPS stimulation, TNF-α release decreased with increasing substrate rigidity. However, gene expression did not readily predict the observed changes in phagocytosis and inflammatory cytokine release associated with differing substrate rigidity. | (Patel et al., 2012) |
| Stiffness (0.3, 1, 6, 27, 47, 120, 230 kPa) | Polyacrylamide | Poly-L-lysine, laminin or collagen | Mouse bone marrow-derived macrophages/2D |  |  | 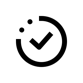 |  | TNF-α, IL-1β, and NO levels increased as substrate stiffness increased without activation. Stimulated macrophages grown on PA gels increased TNF-α, IL-1β, IL-6, and NO concentrations as substrate stiffness increased. This stiffness–regulated proinflammatory mediator production occurs when gels are functionalized with or without ECM. Stiff substrates enhanced TLR4 and MyD88 expression, IκBα phosphorylation, and NF-κB p65 phosphorylation and translocation. | (Previtera and Sengupta, 2015) |
| Stiffness (27, 58, 74, 119 Pa) | Collagen I | Intrinsic domain | Human monocyte-derived macrophage/3D | 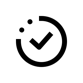 | 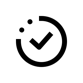 | 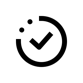 |  | Macrophages are instructed to polarize toward an anti‐inflammatory phenotype (low TNFα, IL‐12, and high IL‐10) by stiffer 3D matrices even under inflammatory conditions (GM‐CSF and LPS). The presence of both GAGs, nonsulfated and sulfated HA, inhibited this stiffness‐mediated effect. | (Friedemann et al., 2017) |
| Stiffness (4, 15, 100 kPa) | Agarose | NA | THP-1/2D | 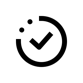 | 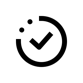 |  | 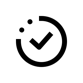 | Macrophages on the 1% gels induced minimum up-regulation of CD64 but marked up-regulation of CD206 and had suppressed ability to internalize beads. Microarray analysis shows that the soft substrate down-regulated M1-related genes such as TNF-α, MCP-1, IL-6, and IRF5, but up-regulated M2-related genes such as PPARγ and VEGFA | (Okamoto et al., 2018) |
| Stiffness (0.5, 1, 1.5 kPa) | Collagen | Intrinsic domain | THP-1/2D |  | 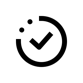 | 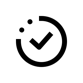 |  | When exposed to scaffolds of similar bulk modulus and degradation characteristics cross-linked using different cross-linkers, the cells responded to the cross-linking agent used rather than to the bulk modulus of the scaffolds. While genipin cross-linking suppressed IL6 level upon addition of M1(IFN-γ +LPS) and reduced IL-10 and IL-1Ra expression after M2(IL-4+IL-13) induction, EDAC cross-linking promoted TNF-α and MIP1α expression in response to M1 factors and production of anti-inflammatory proteins (IL-10 and IL-1Ra) and genes (CCL13 and CCL22) upon M2 activation. | (Sridharan et al., 2019b) |
| Stiffness (11, 88, 323 kPa) | Polyacrylamide | Collagen | THP-1/2D |  | 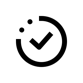 | 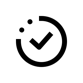 | 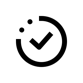 | Stiff polyacrylamide gels prime macrophages towards high TNF-α, IL-6 and MIP-1α secretion, upregulated CCL20 expression with impaired phagocytosis in macrophages, while soft and medium stiffness gels prime cells towards high IL-10 secretion, upregulated CCL13 and CCL117 expression, highly phagocytic phenotype. On soft and medium stiffness gels, cells display Rho-A kinase-dependent, podosome-independent fast amoeboid migration and on stiff gels they adopt a ROCK-independent, podosome-dependent slow mesenchymal migration mode. | (Sridharan et al., 2019a) |
| Stiffness (100, 900, 2500 Pa) | Poly(ethylene glycol) diacrylate | NA | Volumetric muscle loss surgery in mice | 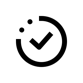 |  |  |  | A decrease in MHCII and CD206 expression correlated with increasing material stiffness. | (Sadtler et al., 2019) |
| Stiffness (2.5, 35, 63.5 kPa) | Polyacrylamide | NA | Mouse bone marrow-derived macrophages/2D | 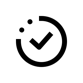 | 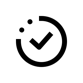 | 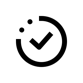 |  | Macrophages cultured on the soft surface displayed enhanced expression of CD86 and production of reactive oxygen species, and secreted more IL-1β and TNF-α. On medium stiffness matrix, macrophages expressed more CD206, produced less ROS, and secreted more IL-4 and TGF-β. The expressions of NIK, phosphorylated p65 and phosphorylated IκB were significantly increased after stimulation with low stiffness in macrophages. | (Chen et al., 2020) |
|  |  |  | Subcutaneous implantation in mice | 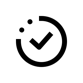 |  |  |  | More CD68+CD86+ cells around the hydrogels with the low substrate stiffness while more CD68+CD206+ cells near by the middle stiffness hydrogels. |  |
| Stiffness (2, 10, 29 kPa) | Gelatin | Intrinsic domain | Mouse bone marrow-derived macrophages/2D |  |  | 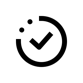 |  | iNOS was more expressed by macrophages on stiff and medium hydrogel matrixes than on soft hydrogels. Arg-1 was mostly expressed by macrophages on soft hydrogels. Also, an increased TNF-α and IL-6 secretion of macrophages and decreased TGF-β and CCL17 on stiff hydrogel surfaces after 3 days of culture was observed. | (Zhuang et al., 2020) |
|  |  |  | Subcutaneous implantation in mice |  |  |  | 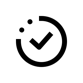 | A significantly larger number of macrophages were infiltrated into the soft hydrogels. A higher percentage of Arg-1 positive cells and lower percentage of iNOS positive cells were found on soft hydrogels. The fibrous tissue formed by soft hydrogels was also significantly thinner. |  |
| Surface topography (roughness) | Titanium | NA | RAW 264.7/2D |  |  | 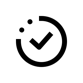 |  | Unstimulated macrophages increased their secretion of TNF‐α when attached to rough surfaces. In macrophages stimulated with LPS, the roughest surface produced higher levels of IL‐1β, IL‐6, and TNF‐α at 24 and 48 h than all other surfaces. | (Refai et al., 2004) |
| Surface topography (roughness) | Titanium | NA | J744A.1/2D |  | 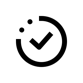 | 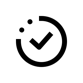 |  | Without activation, IL-1β levels were increased on grit-blasted/acid rough surfaces during the first 48 h while IL-6 levels were reduced. When treated with LPS, high levels of IL-1β and IL-6 expression were measured irrespective of surface topography. There was an LPS dose-dependent enhancement of NO production on both smooth and rough surfaces. | (Tan et al., 2006) |
| Substrate topography (microstructured or nanotexured) | Poly(vinylidene) fluoride | NA | Human peripheral blood mononuclear cells/2D | 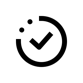 | 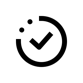 | 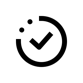 |  | The cytokine pattern elicited by the microstructure was predominantly proinflammatory with typical inflammatory mediators (e.g., IL-1 and IL-6) induced but anti-inflammatory cytokines unchanged or downregulated (IL-10 and IL-1RN). The microstructure seems to cause a phenotype with proinflammatory properties but not the same with the classically activated subtype induced by LPS. | (Paul et al., 2008) |
| Substrate topography (250 nm-2 μm, Nano- and microgratings) | Poly(epsilon-caprolactone), poly(lactic acid) and poly(dimethyl siloxane) | NA | RAW 264.7/2D |  |  | 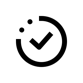 |  | Reduced TNF‐α and VEGF secretion levels on larger grating sizes at 48 hours but the overall patterns in topography-induced cytokine secretion were difficult to discern. | (Chen et al., 2010) |
|  |  |  | Subcutaneous implantation in rat |  |  |  | 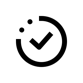 | Reduced macrophage adhesion density and degree of high cell fusion on 2 μm gratings compared to planar controls. |  |
| Surface topography (roughness) | Titanium | NA | RAW 264.7/2D | 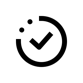 |  | 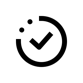 |  | Substrate topography can activate the NF-κB-mediated inflammatory signaling pathway via cell surface receptors found in actin-associated, lipid-enriched rafts on the cell membrane | (Waterfield et al., 2010) |
| Surface topography (matrix architecture) | Collagen I or Matrigel | Intrinsic domain | Human peripheral blood mononuclear cells/3D |  |  |  | 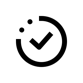 | Macrophages use either the amoeboid migration mode in fibrillar collagen I or the mesenchymal migration mode in Matrigel and gelled collagen I. Macrophages infiltrating matrices of similar composition but with variable stiffness adapt their migration mode primarily to the matrix architecture. | (Van Goethem et al., 2010) |
| Surface topography (fiber diameter and alignment) | Poly(L-lactic) | NA | RAW 264.7/2D |  |  | 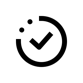 |  | Reduction in the levels of TNF-α, IFN-γ and VEGF was more significant on nanofibrous scaffolds (both random and aligned) compared with PLLA films and microfibrous scaffolds (both random and aligned); a higher number of FBGCs were observed on the PLLA film than on the micro- and nanofibrous scaffolds. | (Saino et al., 2011) |
| Substrate topography (10-200 nm nanodot arrays) | Titanium | NA | Mouse peritoneal macrophage/2D |  | 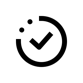 |  |  | Macrophage attachment was higher on surfaces with 10–50 nm features than on flat surfaces and lower on 100-200 nm nanotopographies. Also, pro-inflammatory genes like IL-6 and CCL-3 were mildly up-regulated on surfaces of 100-200 nm features. | (Mohiuddin et al., 2012) |
| Surface topography (roughness) | Titanium | NA | RAW 264.7/2D |  | 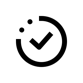 | 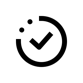 |  | Rough SLA surfaces did not activate Arg‐1 and NOS2 expression, but relative to Po surfaces MCP‐1 and MIP‐1α were upregulated after 5 days, whereas the secretion of CXCL‐10 was lowered. | (Barth et al., 2013) |
| Substrate topography (20 and 50 μm microgratings) | PDMS | Fibronectin | Mouse bone marrow-derived macrophages/2D | 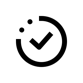 |  | 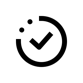 |  | The elongation of macrophages led to higher levels of Arg-1, CD206, and Chil3, although elongation had no effect on the expression of iNOS and the secretion of proinflammatory cytokines was reduced. | (McWhorter et al., 2013) |
| Surface topography (fiber alignment) | Polylactic acid or chitosan | Intrinsic domain | Human peripheral blood mononuclear cells/3D |  |  | 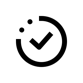 |  | PLA-based scaffolds induced higher production of IL-6, IL-12/23 and IL-10, while Ch led to increased secretion of TNF-α; the orthogonal scaffolds stimulating higher levels of TNF-α and IL-12/23, particularly for late time points, correlating with an increase in the metabolic activity. | (Almeida et al., 2014) |
| Substrate topography (micro- and nanopatterned grooves) | Titanium | NA | Mouse bone marrow-derived macrophages/2D | 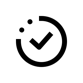 |  | 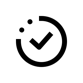 |  | At very small groove widths, expression of Arg-1 was low, and increasing the groove width led to an increase in Arg-1 expression peaking broadly on substrates containing 400 nm to 5 μm grooves. The expression of iNOS remained similarly low across all groove widths. While secretion of TNF-α remained low in macrophages across all conditions, macrophages secreted significantly higher levels of IL-10 on intermediate groove widths compared to cells on other Ti surfaces. | (Luu et al., 2015) |
| Substrate topography (microwell) | PDMS | Fibronectin | Mouse bone marrow-derived macrophages/2D |  |  | 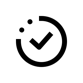 |  | The most elongated single macrophages, assuming the 1:10 aspect ratio, secreted the least amount of MCP-1, whereas the roundest macrophages (1:1) secreted the most MCP-1. | (McWhorter et al., 2016) |
| Substrate topography (microgratings) | Polyethylene | NA | Mouse bone marrow-derived macrophages/2D | 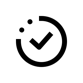 |  | 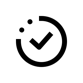 |  | Macrophages elongate along the direction of the uniaxial wrinkles made from shape memory polymers, and express more Arg-1 and IL-10, and less TNF-α. | (Wang et al., 2016) |
|  |  |  | Subcutaneous implantation in mice |  |  |  | 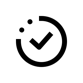 | Material surface topography altered the distribution of collagen deposition in the adjacent tissue, with denser collagen tissue observed near flat materials when compared to wrinkled materials; cells surrounding wrinkled materials exhibited higher Arg-1 expression. |  |
| Substrate topography (convex and concave) | PDMS | NA | THP1/2D | 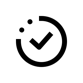 |  | 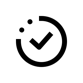 | 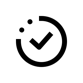 | Macrophage shape and size was different in concave and convex surfaces, but no correlation was found with the cell polarization state. | (Malheiro et al., 2016) |
| Surface topography (roughness and hydrophilicity) | Polystyrene | NA | Human peripheral blood mononuclear cells/2D |  |  | 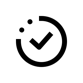 |  | When surface hydrophilicity was increased through plasma gas treatment, there were significant changes in MRC1, IL-10, CCL18, CXCL10, CXCL11 expression in unstimulated macrophages. In the presence of IL-4 or IFN-γ, surface topography had no significant effect on expression of either M1 or M2 associated genes. However, increased hydrophilicity led to an increase in expression of CXCL10 and CXCL11. Similarly, for macrophages polarized to an M2 phenotype with IL-4, cultured on both of the hydrophilic, plasma-etched surfaces increased expression of IL-10, CCL18. | (Kosoff et al., 2018) |
| Geometry (average intranodal distance of 1.2, 3, 4.4 µm) | polytetrafluoroethylene | NA | Human peripheral blood mononuclear cells/2D |  | 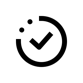 | 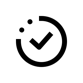 |  | Macrophages cultured on 1.2-ePTFE and 3.0-ePTFE stimulated intermediate levels of IL-1β, and 4.4-ePTFE stimulated a 15-fold increase over np-PTFE. The cDNA and gene expression of IL-1β, IL-6, TNF-α, MCP-1 and MIP1-β were at higher levels by macrophages cultured on 4.4-ePTFE. | (Bota et al., 2010) |
|  |  |  | Subcutaneous implantation in mice |  |  |  | 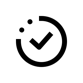 | Subcutaneous implantation of 4.4-ePTFE into mice resulted in a statistically thinner capsule that appeared less organized and less dense than the np-PTFE response. |  |
| Geometry (pore sizes 20, 30, 60 μm) | Poly(2-hydroxyethyl methacrylate-co-methacrylic acid) | NA | Subcutaneous implantation in rat |  |  |  | 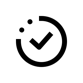 | Cardiac implantation of acellular scaffolds with pore diameters of 30-40 µm showed angiogenesis and reduced fibrotic response, coinciding with a shift in macrophage phenotype toward the M2 state (MMR+). | (Madden et al., 2010) |
| Geometry (1, 11, 15 µm pore size and 0.35, 2.2, 2.8 µm fiber diameter) | Polydioxanone | NA | Mouse bone marrow-derived macrophages/2D |  | 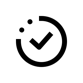 | 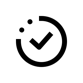 |  | A correlation between increasing fiber/pore size and increased expression of Arg-1, along with decreased expression of iNOS; secretion of VEGF, TGF-β1 and bFGF was higher among cultures employing larger fiber/pore size scaffolds; a potential role for MyD88 in regulating M1 macrophages signaling on the large fiber/pore size PDO scaffold comparing to the small one. | (Garg et al., 2013) |
| Geometry (pore sizes 34, 160 μm) | Poly(2-hydroxyethyl methacrylate) | NA | Subcutaneous implantation in mice | 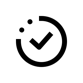 |  |  |  | Macrophages in 34 μm porous implants had up to 63% greater expression of iNOS and IL-1R1 and up to 85% reduction in MMR and SR-BI/II expression. | (Sussman et al., 2014) |
| Geometry (pore sizes 4.6, 41 μm) | Poly(ε-caprolactone) | NA | RAW 264.7/2D |  |  | 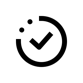 |  | Expression of IL-6, TNF-α and MIF-1 was evidently elevated in cells cultured on the thinner-fiber at day 3, whereas Arg-1 and Fizz1 were highly expressed in cells on the thicker fiber. | (Wang et al., 2014) |
|  |  |  | Abdominal aorta implantation in rat | 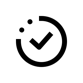 |  |  |  | The thicker fiber scaffolds induced a large number of CD206+ macrophages to infiltrate into the graft wall. |  |
| Geometry (0.5-mm and 1.5-mm spheres) | SLG20 alginate | NA | Subcutaneous implantation in mice |  |  | 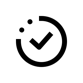 |  | Expression of macrophage markers associated only with classical and wound-healing phenotypes was enriched in 1.5-mm spheres but not regulatory phenotype. In 0.5-mm spheres there was increasing expression of markers associated with all three macrophage phenotypes. | (Veiseh et al., 2015) |
| Geometry (pore sizes 20–30 µm and 355-425 µm) | PDMS | Fibronectin | RAW 264.7 and mouse bone marrow-derived macrophages/2D |  | 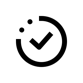 | 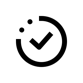 | 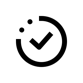 | Preventing macrophage spreading by spatial confinement, either through micropatterning, microporous substrates or cell crowding, can suppress late LPS-activated transcriptional programs (e.g., lower IL-6, CXCL9, IL-1β, and iNOS gene expression, reduced bacterial phagocytosis, decreased TNF-α, IL-6 and IL-12 secretion) by mechanomodulating chromatin compaction and epigenetic alterations (HDAC3 levels and H3K36-dimethylation). | (Jain and Vogel, 2018) |
| Geometry (2 or 6 μm isotropic or anisotropic fibers) | Poly-ε-caprolactone bisurea | NA | THP1/2D |  | 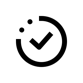 | 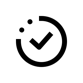 |  | The highest level of ROS-induced lipid peroxidation, NADPH oxidase gene expression and degradation were observed in the 6 μm scaffolds. | (Wissing et al., 2019) |
| Geometry (pore sizes 40, 50, 60, 80, 100 μm) | Poly(ε-caprolactone) | NA | Human peripheral blood mononuclear cells/3D | 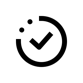 | 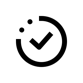 | 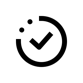 |  | M2-specific markers (CD163, CD206, and IL-10) were higher expressed and released on scaffolds when using smaller pores and decreased with increasing pore size. Gene expression of IL-1β and IL-8 was strongly downregulated on all square porous scaffolds with significant differences to the 2D control. IL-10 was released in significantly higher amounts especially for macrophages cultured on box-shaped scaffolds with a 40 μm pores. | (Tylek et al., 2020) |
| Geometry (pore sizes 160 and 360 μm) | Collagen/chitosan | Intrinsic domain | RAW 264.7/2D |  | 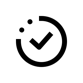 | 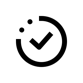 |  | M0 or M1 macrophages seeded on Col-Ch-360 expressed more M2-related genes (TGF-β or PDGF at days 1, 3 or 7) while those seeded on Col-Ch-160 scaffolds tend to express more M1-related genes (IL-1β and IL-6). M0 or M1 cells seeded on Col-Ch-360 scaffolds secrete more M2-related cytokines (TGF-β, IL-10 or IGF) and less M1-related cytokines (IL-6) than those seeded on Col-Ch-160 scaffolds. | (Yin et al., 2020) |
|  |  |  | Subcutaneous implantation in mice | 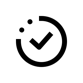 |  |  |  | During the process of vascularization in scaffolds, fewer CCR7+ cells were observed in Col-Ch-360, especially at days 7, 14, and 28, which partly parallels the expression of VEGF+ cells. However, no more CD206+ cells were found in Col-Ch 360 scaffolds |  |
| Surface chemistry (hydrophobic, hydrophilic, and/or ionic chemistries) | Polyethylene terephthalate | NA | Human peripheral blood mononuclear cells/2D |  |  | 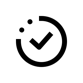 |  | With or without IL-4 activation, hydrophilic and anionic surface increased IL-10 expression and cell adhesion. Cationic surface caused a decrease in IL-10 and cell adhesion. | (Brodbeck et al., 2002) |
| Surface chemistry (hydrophobic/hydrophilic) | Polycarbonate urethane or PDMS | NA | Human peripheral blood mononuclear cells/2D | 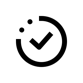 |  |  |  | MMR expression was up-regulated on the silicone-modified polyurethanes and PDMS control in the presence of IL-4. | (Dadsetan et al., 2004) |
| Surface chemistry (hydrophobic, hydrophilic, and/or ionic chemistries) | Polyethylene terephthalate | NA | Human peripheral blood mononuclear cells/2D |  |  | 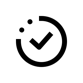 |  | The hydrophilic/neutral surfaces showed a significant decrease in cellular density and minimal FBGC formation, but the adherent cells were further activated to produce significantly greater amounts of IL-10 and less IL-1β and IL-6. | (Jones et al., 2007) |
| Adhesive substrate (integrin engagement) | Polyethylene glycol | RGD, PHSRN, and PRRARV domains | Human peripheral blood mononuclear cells/2D |  |  |  | 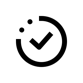 | RGD or PHSRN alone did not provide an adequate substrate for macrophage fusion to form FBGCs. However, the PHSRN synergistic site and the RGD site in a single oligopeptide provided a substrate for FBGC formation that was statistically comparable to that on the positive control material in the presence of serum proteins. This response was highly dependent upon the relative orientation between RGD and PHSRN. PRRARV did not support FBGC formation. | (Kao et al., 2001) |
| Adhesive substrate (integrin engagement) | Poly(ethylene glycol) diacrylate | RGD | Mouse bone marrow-derived macrophages/2D |  |  | 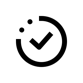 |  | Significantly higher gene expressions of TNF‐α and Il‐1β were found in macrophages seeded onto PEG compared to PEG‐RGD and silicone at 1 and 2 days. | (Lynn et al., 2010) |
|  |  |  | Subcutaneous implantation in mice |  |  |  | 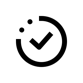 | PEG‐RGD hydrogels resulted in a FBR similar to silicone, while PEG‐only hydrogels resulted in a robust inflammatory reaction with a thick layer of macrophages at the material surface and evidence of gel degradation. |  |
| Adhesive substrate (integrin engagement) | Plastic | Anti‐αVβ3 integrin mAb | Human peripheral blood mononuclear cells/2D |  | 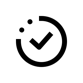 | 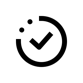 |  | αVβ3 ligation results in sustained increases of the transcription factor NF‐κB DNA‐binding activity. Activation of NF‐κB parallels the increase of TNF‐α, IL‐1β, IL‐6, IL‐8, and a decrease in IL‐10. Upon ligation of the αVβ3 receptor, treatment with TNF‐α or LPS results in the enhanced and synergistic activation of NF‐κB and LPS‐induced TNF‐α secretion. | (Antonov et al., 2011) |
| Adhesive substrate (integrin engagement) | Polyethylene terephthalate | Fibronectin | Subcutaneous implantation in mice |  |  |  | 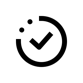 | Mac1 knockout mice displayed reduced cytokine secretion compared to the wild type controls and had reduced fibrous capsule thickness by 27%. Similarly, blocking RGD ligands by releasing a high affinity RGD peptide decreased the fibrous capsule thickness by 45%. | (Zaveri et al., 2014) |
| Adhesive substrate (integrin engagement) | Polystyrene | RGD, vitronectin and chitosan | Human peripheral blood mononuclear cells/2D |  |  | 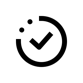 |  | RGD, vitronectin and chitosan adsorbed surfaces stimulated CD147, CD98, CD206 and CD13 expression. | (McNally and Anderson, 2015) |
| Adhesive substrate (integrin engagement) | PDMS | Fibronectin | Mouse bone marrow-derived macrophages/2D |  |  | 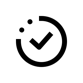 |  | There was a higher single macrophage secretion of MCP-1 on fibronectin than Pluronics. | (McWhorter et al., 2016) |
| Adhesive substrate (integrin engagement) | Gelatin methacryloyl and poly(ethylene glycol) diacrylate | Intrinsic domain or NA | THP1/3D | 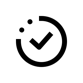 | 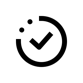 | 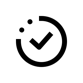 |  | THP‐1 cells encapsulated in GelMA demonstrated significantly higher levels of IL‐1β, IL‐6, IL‐16, IL‐1RA and IL‐10. GelMA hydrogels also induced an increase in monocyte expression of focal adhesion kinase, vinculin, collagen matrix‐related integrin receptors integrin α2 and β1. Blocking integrin α2β1 strongly increased CD86 expression and reduced CD206 expression below the detection limit, even in the presence of IL‐4. | (Cha et al., 2017) |
| Adhesive substrate (integrin engagement) | Laminin, Matrigel, vitronectin, collagen, fibronectin, or fibrinogen | Intrinsic domain | Mouse bone marrow-derived macrophages/2D | 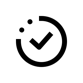 |  | 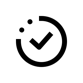 |  | Expression of Arg-1 was enhanced when cells were cultured on laminin, Matrigel, and vitronectin when compared to collagen, fibronectin, or fibrinogen. | (Luu and Liu, 2018) |
| Cyclic pressure (0.138 Mpa, 0.05 or 0.5 Hz) | Polystyrene plastic | NA | Human peripheral blood mononuclear cells/2D |  |  | 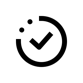 |  | Both regimes of cyclic pressure were found to increase expression of IL-6 and TNF-α. Expression of IL-1β was increased by a higher-frequency regime only. | (Ferrier et al., 2000) |
| Cyclic strains (4% strain at 1 Hz) | Elastomer membranes | Fibronectin | Human peripheral blood mononuclear cells/2D |  |  | 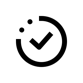 |  | In the presence of phorbol myristate acetate, strain increased MMP-1 expression by 5.1 ± 0.7-fold and MMP-3 expression by 1.6 ± 0.1-fold. | (Yang et al., 2000) |
| Cyclic strains (static or cyclic strain) | BioFlex well | Collagen type I | Mouse peritoneal macrophage/2D | 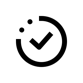 |  | 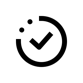 |  | Static stretch increased macrophage expression of inflammatory genes including iNOS, cyclooxygenase-2, IL-1β, IL-6, MIP-1α, and MIP-2. | (Wehner et al., 2010) |
| Cyclic strains (0.5 Hz, 8–20%) | Elastomer membranes | NA | Mouse alveolar macrophages/2D |  |  | 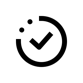 |  | Cyclic stretch activates the nucleotide-binding oligomerization domain-like receptor containing pyrin domain 3 inflammasomes and induces the release of IL-1β in mouse alveolar macrophages via caspase-1- and TLR4-dependent mechanisms. | (Wu et al., 2013) |
| Cyclic strains (0%, 7%, 12% strain) | Polycaprolactone bis-urea | NA | Human peripheral blood mononuclear cells/3D | 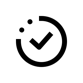 | 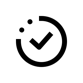 |  |  | Moderate strains (7%) elicit polarization towards a reparative M2 profile and enhance the expression of genes participating in the immune response, such as MCP-1, IL6, IL10, MMP-9, while higher strains (12%) elicit a downregulation of these genes. | (Ballotta et al., 2014) |
| Cyclic strains (0%, 8%, 14% strain) | Ureidopyrimidinone-modified polycaprolactone | NA | Human peripheral blood mononuclear cells/3D | 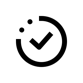 | 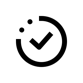 | 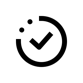 |  | A substantial fraction of cells exhibited the macrophage marker CD86, with no detectable differences between different fiber diameters and strain levels; similar amounts of M1 phenotype and M2 phenotypes were observed in presence of 0% and 8% strain, with frequent colocalization of CCR7 and CD163 on a single cell; qPCR results showed strain-dependent expression of anti- and proinflammatory genes and ECM-related genes; the proinflammatory cytokines IL-6, TNF-α, and IL-1β showed similar trends, with a strain-dictated increase in secretion levels; the deposition of ECM components by macrophages, such as soluble α-elastin, and collagen I and III, is also strain-dependent | (Bonito et al., 2019) |
| Cyclic strains (shear stress ∼1 Pa, cyclic stretch ∼1.04 or a combination) | Polycaprolactone bis-urea | Fibronectin | THP1/3D |  |  |  |  | Exposure to mechanical loading resulted in significant increases in MCP-1, TNF-α and IL-10 gene expression when compared to the statically cultured controls, especially in conditions of shear stress (MCP-1) and cyclic stretch (TNF-α and IL-10); a decrease in pro-inflammatory CCR7 gene expression was detected in the presence of shear stress; elevated levels of MCP-1, IL-10, IL-6, TNF-α, and PDGF-BB secretion were measured upon hemodynamic loading, especially in the combined group, while TGF-β expression was inhibited upon mechanical loading; cyclic stretch decreased macrophage-driven biomaterial degradation | (Wissing et al., 2020) |
| Interstitial flow (3 μm/s) | Collagen I | Intrinsic domain | Mouse bone marrow-derived macrophages or RAW264.7/3D |  |  |  |  | Interstitial flow polarizes macrophages toward an M2-like phenotype by up-regulating the expression of Arg-1, TGF-β, CD206, CD163, and transglutaminase 2 via integrin/Src-mediated mechanotransduction pathways involving STAT3/6. | (Li et al., 2018) |

Abbreviations are as follows: TNF-α, tumor necrosis factor-alpha; IL, interleukins; LPS, lipopolysaccharides; TLR4, toll-like receptor 4; MyD88, myeloid differentiation primary response 88; IκBα, nuclear factor of kappa light polypeptide gene enhancer in B-cells inhibitor, alpha; NF-κB, nuclear factor kappa-light-chain-enhancer of activated B cells; M-CSF, macrophage-colony stimulating factor; GM-CSF, granulocyte macrophage colony stimulating factor; GAGs, glycosaminoglycans; MCP-1, monocyte chemoattractant protein-1; IRF5, interferon regulatory factor 5; PPARγ, peroxisome proliferator-activated receptor; VEGF, vascular endothelial growth factor; MIP-1α, macrophage inflammatory protein 1-alpha; CXCL, chemokine (C-X-C motif) ligand; CCL, chemokine ligand; MHCII, major histocompatibility complex class II; iNOS, inducible nitric oxide synthase; Arg-1, arginase-1; NOS2, nitric oxide synthase 2; Chil3, chitinase-like 3; MMR, macrophage mannose receptor; bFGF, basic fibroblast growth factor; SR-BI/II, scavenger receptor BI and BII; Fizz1, resistin-like molecule alpha1; HDAC3, histone deacetylase 3; H3K36, H3 lysine 36; NADPH, nicotinamide adenine dinucleotide phosphate; PDGF, platelet-derived growth factor; IGF, insulin-like growth factor; CCR, CC chemokine receptors; MMP, matrix metalloproteinase.

# Reference

Almeida, C.R., Serra, T., Oliveira, M.I., Planell, J.A., Barbosa, M.A., and Navarro, M. (2014). Impact of 3-D printed PLA- and chitosan-based scaffolds on human monocyte/macrophage responses: Unraveling the effect of 3-D structures on inflammation. *Acta Biomaterialia* 10(2)**,** 613-622. doi: 10.1016/j.actbio.2013.10.035.

Antonov, A.S., Antonova, G.N., Munn, D.H., Mivechi, N., Lucas, R., Catravas, J.D., et al. (2011). alphaVbeta3 integrin regulates macrophage inflammatory responses via PI3 kinase/Akt-dependent NF-kappaB activation. *J Cell Physiol* 226(2)**,** 469-476. doi: 10.1002/jcp.22356.

Ballotta, V., Driessen-Mol, A., Bouten, C.V.C., and Baaijens, F.P.T. (2014). Strain-dependent modulation of macrophage polarization within scaffolds. *Biomaterials* 35(18)**,** 4919-4928. doi: 10.1016/j.biomaterials.2014.03.002.

Barth, K.A., Waterfield, J.D., and Brunette, D.M. (2013). The effect of surface roughness on RAW 264.7 macrophage phenotype. *J Biomed Mater Res A* 101(9)**,** 2679-2688. doi: 10.1002/jbm.a.34562.

Blakney, A.K., Swartzlander, M.D., and Bryant, S.J. (2012). The effects of substrate stiffness on the in vitro activation of macrophages and in vivo host response to poly(ethylene glycol)-based hydrogels. *J Biomed Mater Res A* 100(6)**,** 1375-1386. doi: 10.1002/jbm.a.34104.

Bonito, V., de Kort, B.J., Bouten, C.V.C., and Smits, A. (2019). Cyclic Strain Affects Macrophage Cytokine Secretion and Extracellular Matrix Turnover in Electrospun Scaffolds. *Tissue Eng Part A* 25(17-18)**,** 1310-1325. doi: 10.1089/ten.TEA.2018.0306.

Bota, P.C., Collie, A.M., Puolakkainen, P., Vernon, R.B., Sage, E.H., Ratner, B.D., et al. (2010). Biomaterial topography alters healing in vivo and monocyte/macrophage activation in vitro. *J Biomed Mater Res A* 95(2)**,** 649-657. doi: 10.1002/jbm.a.32893.

Brodbeck, W.G., Nakayama, Y., Matsuda, T., Colton, E., Ziats, N.P., and Anderson, J.M. (2002). Biomaterial surface chemistry dictates adherent monocyte/macrophage cytokine expression in vitro. *Cytokine* 18(6)**,** 311-319. doi: 10.1006/cyto.2002.1048.

Cha, B.H., Shin, S.R., Leijten, J., Li, Y.C., Singh, S., Liu, J.C., et al. (2017). Integrin-Mediated Interactions Control Macrophage Polarization in 3D Hydrogels. *Adv Healthc Mater* 6(21)**,** 1700289. doi: 10.1002/adhm.201700289.

Chen, M., Zhang, Y., Zhou, P., Liu, X., Zhao, H., Zhou, X., et al. (2020). Substrate stiffness modulates bone marrow-derived macrophage polarization through NF-kappaB signaling pathway. *Bioact Mater* 5(4)**,** 880-890. doi: 10.1016/j.bioactmat.2020.05.004.

Chen, S., Jones, J.A., Xu, Y., Low, H.Y., Anderson, J.M., and Leong, K.W. (2010). Characterization of topographical effects on macrophage behavior in a foreign body response model. *Biomaterials* 31(13)**,** 3479-3491. doi: 10.1016/j.biomaterials.2010.01.074.

Dadsetan, M., Jones, J.A., Hiltner, A., and Anderson, J.M. (2004). Surface chemistry mediates adhesive structure, cytoskeletal organization, and fusion of macrophages. *J Biomed Mater Res A* 71(3)**,** 439-448. doi: 10.1002/jbm.a.30165.

Ferrier, G.M., McEvoy, A., Evans, C.E., and Andrew, J.G. (2000). The effect of cyclic pressure on human monocyte-derived macrophages in vitro. *J Bone Joint Surg Br* 82(5)**,** 755-759. doi: 10.1302/0301-620x.82b5.9561.

Friedemann, M., Kalbitzer, L., Franz, S., Moeller, S., Schnabelrauch, M., Simon, J.C., et al. (2017). Instructing Human Macrophage Polarization by Stiffness and Glycosaminoglycan Functionalization in 3D Collagen Networks. *Adv Healthc Mater* 6(7)**,** 1600967. doi: 10.1002/adhm.201600967.

Garg, K., Pullen, N.A., Oskeritzian, C.A., Ryan, J.J., and Bowlin, G.L. (2013). Macrophage functional polarization (M1/M2) in response to varying fiber and pore dimensions of electrospun scaffolds. *Biomaterials* 34(18)**,** 4439-4451. doi: 10.1016/j.biomaterials.2013.02.065.

Irwin, E.F., Saha, K., Rosenbluth, M., Gamble, L.J., Castner, D.G., and Healy, K.E. (2008). Modulus-dependent macrophage adhesion and behavior. *J Biomater Sci Polym Ed* 19(10)**,** 1363-1382. doi: 10.1163/156856208786052407.

Jain, N., and Vogel, V. (2018). Spatial confinement downsizes the inflammatory response of macrophages. *Nat Mater* 17(12)**,** 1134-1144. doi: 10.1038/s41563-018-0190-6.

Jones, J.A., Chang, D.T., Meyerson, H., Colton, E., Kwon, I.K., Matsuda, T., et al. (2007). Proteomic analysis and quantification of cytokines and chemokines from biomaterial surface-adherent macrophages and foreign body giant cells. *J Biomed Mater Res A* 83(3)**,** 585-596. doi: 10.1002/jbm.a.31221.

Kao, W.J., Lee, D., Schense, J.C., and Hubbell, J.A. (2001). Fibronectin modulates macrophage adhesion and FBGC formation: the role of RGD, PHSRN, and PRRARV domains. *J Biomed Mater Res* 55(1)**,** 79-88. doi: 10.1002/1097-4636(200104)55:1<79::aid-jbm110>3.0.co;2-z.

Kosoff, D., Yu, J., Suresh, V., Beebe, D.J., and Lang, J.M. (2018). Surface topography and hydrophilicity regulate macrophage phenotype in milled microfluidic systems. *Lab Chip* 18(19)**,** 3011-3017. doi: 10.1039/c8lc00431e.

Li, R., Serrano, J.C., Xing, H., Lee, T.A., Azizgolshani, H., Zaman, M., et al. (2018). Interstitial flow promotes macrophage polarization toward an M2 phenotype. *Mol Biol Cell* 29(16)**,** 1927-1940. doi: 10.1091/mbc.E18-03-0164.

Luu, T.U., Gott, S.C., Woo, B.W., Rao, M.P., and Liu, W.F. (2015). Micro- and Nanopatterned Topographical Cues for Regulating Macrophage Cell Shape and Phenotype. *ACS Appl Mater Interfaces* 7(51)**,** 28665-28672. doi: 10.1021/acsami.5b10589.

Luu, T.U., and Liu, W.F. (2018). Regulation of Macrophages by Extracellular Matrix Composition and Adhesion Geometry. *Regenerative Engineering and Translational Medicine* 4(4)**,** 238-246. doi: 10.1007/s40883-018-0065-z.

Lynn, A.D., Kyriakides, T.R., and Bryant, S.J. (2010). Characterization of the in vitro macrophage response and in vivo host response to poly(ethylene glycol)-based hydrogels. *J Biomed Mater Res A* 93(3)**,** 941-953. doi: 10.1002/jbm.a.32595.

Madden, L.R., Mortisen, D.J., Sussman, E.M., Dupras, S.K., Fugate, J.A., Cuy, J.L., et al. (2010). Proangiogenic scaffolds as functional templates for cardiac tissue engineering. *Proc Natl Acad Sci U S A* 107(34)**,** 15211-15216. doi: 10.1073/pnas.1006442107.

Malheiro, V., Lehner, F., Dinca, V., Hoffmann, P., and Maniura-Weber, K. (2016). Convex and concave micro-structured silicone controls the shape, but not the polarization state of human macrophages. *Biomater Sci* 4(11)**,** 1562-1573. doi: 10.1039/c6bm00425c.

McNally, A.K., and Anderson, J.M. (2015). Phenotypic expression in human monocyte-derived interleukin-4-induced foreign body giant cells and macrophages in vitro: dependence on material surface properties. *J Biomed Mater Res A* 103(4)**,** 1380-1390. doi: 10.1002/jbm.a.35280.

McWhorter, F.Y., Smith, T.D., Luu, T.U., Rahim, M.K., Haun, J.B., and Liu, W.F. (2016). Macrophage secretion heterogeneity in engineered microenvironments revealed using a microwell platform. *Integr Biol (Camb)* 8(7)**,** 751-760. doi: 10.1039/c6ib00053c.

McWhorter, F.Y., Wang, T., Nguyen, P., Chung, T., and Liu, W.F. (2013). Modulation of macrophage phenotype by cell shape. *Proc Natl Acad Sci U S A* 110(43)**,** 17253-17258. doi: 10.1073/pnas.1308887110.

Mohiuddin, M., Pan, H.A., Hung, Y.C., and Huang, G.S. (2012). Control of growth and inflammatory response of macrophages and foam cells with nanotopography. *Nanoscale Res Lett* 7(1)**,** 394. doi: 10.1186/1556-276X-7-394.

Okamoto, T., Takagi, Y., Kawamoto, E., Park, E.J., Usuda, H., Wada, K., et al. (2018). Reduced substrate stiffness promotes M2-like macrophage activation and enhances peroxisome proliferator-activated receptor gamma expression. *Exp Cell Res* 367(2)**,** 264-273. doi: 10.1016/j.yexcr.2018.04.005.

Patel, N.R., Bole, M., Chen, C., Hardin, C.C., Kho, A.T., Mih, J., et al. (2012). Cell elasticity determines macrophage function. *PLoS One* 7(9)**,** e41024. doi: 10.1371/journal.pone.0041024.

Paul, N.E., Skazik, C., Harwardt, M., Bartneck, M., Denecke, B., Klee, D., et al. (2008). Topographical control of human macrophages by a regularly microstructured polyvinylidene fluoride surface. *Biomaterials* 29(30)**,** 4056-4064. doi: 10.1016/j.biomaterials.2008.07.010.

Previtera, M.L., and Sengupta, A. (2015). Substrate Stiffness Regulates Proinflammatory Mediator Production through TLR4 Activity in Macrophages. *PLoS One* 10(12)**,** e0145813. doi: 10.1371/journal.pone.0145813.

Refai, A.K., Textor, M., Brunette, D.M., and Waterfield, J.D. (2004). Effect of titanium surface topography on macrophage activation and secretion of proinflammatory cytokines and chemokines. *J Biomed Mater Res A* 70(2)**,** 194-205. doi: 10.1002/jbm.a.30075.

Sadtler, K., Wolf, M.T., Ganguly, S., Moad, C.A., Chung, L., Majumdar, S., et al. (2019). Divergent immune responses to synthetic and biological scaffolds. *Biomaterials* 192**,** 405-415. doi: 10.1016/j.biomaterials.2018.11.002.

Saino, E., Focarete, M.L., Gualandi, C., Emanuele, E., Cornaglia, A.I., Imbriani, M., et al. (2011). Effect of electrospun fiber diameter and alignment on macrophage activation and secretion of proinflammatory cytokines and chemokines. *Biomacromolecules* 12(5)**,** 1900-1911. doi: 10.1021/bm200248h.

Sridharan, R., Cavanagh, B., Cameron, A.R., Kelly, D.J., and O'Brien, F.J. (2019a). Material stiffness influences the polarization state, function and migration mode of macrophages. *Acta Biomater* 89**,** 47-59. doi: 10.1016/j.actbio.2019.02.048.

Sridharan, R., Ryan, E.J., Kearney, C.J., Kelly, D.J., and O'Brien, F.J. (2019b). Macrophage Polarization in Response to Collagen Scaffold Stiffness Is Dependent on Cross-Linking Agent Used To Modulate the Stiffness. *Acs Biomaterials Science & Engineering* 5(2)**,** 544-552. doi: 10.1021/acsbiomaterials.8b00910.

Sussman, E.M., Halpin, M.C., Muster, J., Moon, R.T., and Ratner, B.D. (2014). Porous implants modulate healing and induce shifts in local macrophage polarization in the foreign body reaction. *Ann Biomed Eng* 42(7)**,** 1508-1516. doi: 10.1007/s10439-013-0933-0.

Tan, K.S., Qian, L., Rosado, R., Flood, P.M., and Cooper, L.F. (2006). The role of titanium surface topography on J774A.1 macrophage inflammatory cytokines and nitric oxide production. *Biomaterials* 27(30)**,** 5170-5177. doi: 10.1016/j.biomaterials.2006.05.002.

Tylek, T., Blum, C., Hrynevich, A., Schlegelmilch, K., Schilling, T., Dalton, P.D., et al. (2020). Precisely defined fiber scaffolds with 40 mum porosity induce elongation driven M2-like polarization of human macrophages. *Biofabrication* 12(2)**,** 025007. doi: 10.1088/1758-5090/ab5f4e.

Van Goethem, E., Poincloux, R., Gauffre, F., Maridonneau-Parini, I., and Le Cabec, V. (2010). Matrix architecture dictates three-dimensional migration modes of human macrophages: differential involvement of proteases and podosome-like structures. *J Immunol* 184(2)**,** 1049-1061. doi: 10.4049/jimmunol.0902223.

Veiseh, O., Doloff, J.C., Ma, M., Vegas, A.J., Tam, H.H., Bader, A.R., et al. (2015). Size- and shape-dependent foreign body immune response to materials implanted in rodents and non-human primates. *Nat Mater* 14(6)**,** 643-651. doi: 10.1038/nmat4290.

Wang, T., Luu, T.U., Chen, A., Khine, M., and Liu, W.F. (2016). Topographical modulation of macrophage phenotype by shrink-film multi-scale wrinkles. *Biomater Sci* 4(6)**,** 948-952. doi: 10.1039/c6bm00224b.

Wang, Z., Cui, Y., Wang, J., Yang, X., Wu, Y., Wang, K., et al. (2014). The effect of thick fibers and large pores of electrospun poly(epsilon-caprolactone) vascular grafts on macrophage polarization and arterial regeneration. *Biomaterials* 35(22)**,** 5700-5710. doi: 10.1016/j.biomaterials.2014.03.078.

Waterfield, J.D., Ali, T.A., Nahid, F., Kusano, K., and Brunette, D.M. (2010). The effect of surface topography on early NFkappaB signaling in macrophages. *J Biomed Mater Res A* 95(3)**,** 837-847. doi: 10.1002/jbm.a.32857.

Wehner, S., Buchholz, B.M., Schuchtrup, S., Rocke, A., Schaefer, N., Lysson, M., et al. (2010). Mechanical strain and TLR4 synergistically induce cell-specific inflammatory gene expression in intestinal smooth muscle cells and peritoneal macrophages. *Am J Physiol Gastrointest Liver Physiol* 299(5)**,** G1187-1197. doi: 10.1152/ajpgi.00452.2009.

Wissing, T.B., Bonito, V., van Haaften, E.E., van Doeselaar, M., Brugmans, M., Janssen, H.M., et al. (2019). Macrophage-Driven Biomaterial Degradation Depends on Scaffold Microarchitecture. *Front Bioeng Biotechnol* 7(87)**,** 87. doi: 10.3389/fbioe.2019.00087.

Wissing, T.B., van Haaften, E.E., Koch, S.E., Ippel, B.D., Kurniawan, N.A., Bouten, C.V., et al. (2020). Hemodynamic loads distinctively impact the secretory profile of biomaterial-activated macrophages–implications for in situ vascular tissue engineering. *Biomaterials Science* 8(1)**,** 132-147.

Wu, J., Yan, Z., Schwartz, D.E., Yu, J., Malik, A.B., and Hu, G. (2013). Activation of NLRP3 inflammasome in alveolar macrophages contributes to mechanical stretch-induced lung inflammation and injury. *J Immunol* 190(7)**,** 3590-3599. doi: 10.4049/jimmunol.1200860.

Yang, J.H., Sakamoto, H., Xu, E.C., and Lee, R.T. (2000). Biomechanical regulation of human monocyte/macrophage molecular function. *Am J Pathol* 156(5)**,** 1797-1804. doi: 10.1016/S0002-9440(10)65051-1.

Yin, Y., He, X.-T., Wang, J., Wu, R.-X., Xu, X.-Y., Hong, Y.-L., et al. (2020). Pore size-mediated macrophage M1-to-M2 transition influences new vessel formation within the compartment of a scaffold. *Applied Materials Today* 18**,** 100466. doi: 10.1016/j.apmt.2019.100466.

Zaveri, T.D., Lewis, J.S., Dolgova, N.V., Clare-Salzler, M.J., and Keselowsky, B.G. (2014). Integrin-directed modulation of macrophage responses to biomaterials. *Biomaterials* 35(11)**,** 3504-3515. doi: 10.1016/j.biomaterials.2014.01.007.

Zhuang, Z.M., Zhang, Y., Sun, S.N., Li, Q., Chen, K.W., An, C.F., et al. (2020). Control of Matrix Stiffness Using Methacrylate-Gelatin Hydrogels for a Macrophage-Mediated Inflammatory Response. *Acs Biomaterials Science & Engineering* 6(5)**,** 3091-3102. doi: 10.1021/acsbiomaterials.0c00295.
